# A Combined Phytochemistry and Network Pharmacology Approach to Reveal Potential Anti-NSCLC Effective Substances and Mechanisms in *Marsdenia tenacissima* (Roxb.) Moon (Stem)

**DOI:** 10.3389/fphar.2021.518406

**Published:** 2021-04-29

**Authors:** Pei Liu, Dong-Wei Xu, Run-Tian Li, Shao-Hui Wang, Yan-Lan Hu, Shao-Yu Shi, Jia-Yao Li, Yu-He Huang, Li-Wei Kang, Tong-Xiang Liu

**Affiliations:** ^1^ School of Pharmacy, Minzu University of China, Beijing, China; ^2^ Key Laboratory of Ethnomedicine (Minzu University of China), Minority of Education, Beijing, China; ^3^ Medical College of Qingdao Binhai University, Affiliated Hospital of Qingdao Binhai University, Qingdao, China

**Keywords:** *Marsdeniae tenacissimae*, C21 steroidal saponins, network pharmacology, migration, invasion, apoptosis, A549 cells, NSCLC

## Abstract

*Marsdeniae tenacissimae* Caulis is a traditional Chinese medicine, named Tongguanteng (TGT), that is often used for the adjuvant treatment of cancer. In our previous study, we reported that an ethyl acetate extract of TGT had inhibitory effects against adenocarcinoma A549 cells growth. To identify the components of TGT with anti-tumor activity and to elucidate their underlying mechanisms of action, we developed a technique for isolating compounds, which was then followed by cytotoxicity screening, network pharmacology analysis, and cellular and molecular experiments. We isolated a total of 19 compounds from a TGT ethyl acetate extract. Two novel steroidal saponins were assessed using an ultra-performance liquid chromatography-photodiode array coupled with quadrupole time-of-flight mass (UPLC-ESI-Q/TOF-MS). Then, we screened these constituents for anti-cancer activity against non-small cell lung cancer (NSCLC) *in vitro* and obtained six target compounds. Furthermore, a compound-target-pathway network of these six bioactive ingredients was constructed to elucidate the potential pathways that controlled anticancer effects. Approximately 205 putative targets that were associated with TGT, as well as 270 putative targets that were related to NSCLC, were obtained from online databases and target prediction software. Protein–protein interaction networks for drugs as well as disease putative targets were generated, and 18 candidate targets were detected based on topological features. In addition, pathway enrichment analysis was performed to identify related pathways, including PI3K/AKT, VEGF, and EGFR tyrosine kinase inhibitor resistance, which are all related to metabolic processes and intrinsic apoptotic pathways involving reactive oxygen species (ROS). Then, various cellular experiments were conducted to validate drug-target mechanisms that had been predicted using network pharmacology analysis. The experimental results showed the four C21 steroidal saponins could upregulate Bax and downregulate Bcl-2 expression, thereby changing the mitochondrial membrane potential, producing ROS, and releasing cytochrome C, which finally activated caspase-3, caspase-9, and caspase-8, all of which induced apoptosis in A549 cells. In addition, these components also downregulated the expression of MMP-2 and MMP-9 proteins, further weakening their degradation of extracellular matrix components and type IV collagen, and inhibiting the migration and invasion of A549 cells. Our study elucidated the chemical composition and underlying anti-tumor mechanism of TGT*,* which may be utilized in the treatment of lung cancer.

## Introduction

Lung cancer severely affects human health and survival and has become the main cause of cancer-related deaths over the past several years (Gu et al., 2018). An estimated 1.6 million individuals have received a diagnosis of lung cancer, resulting in more than 1.3 million deaths worldwide in the past decade (Torre et al., 2015). In 2017, the US pointed out that approximately 85–90% of lung cancer cases were non-small cell lung cancer (NSCLC) (DeSantis et al., 2016; Paci et al., 2017). Over the past 10 years, various major advances in cancer research have uncovered the genetics and pathologies of NSCLC, facilitating the development of novel anticancer drugs (Hare et al., 2017). Interestingly, multiple bioactive ingredients obtained from Chinese herbal medicine have been considered potential candidates for the treatment of cancer (Tang et al., 2009; Gao et al., 2020).

*Marsdeniae tenacissimae* Caulis is the dried lianoid stem of *Marsdenia tenacissima* (Roxb.) Moon (Fam. Asclepiadaceae), known as “Tong-guang-teng” or “Tong-guang-san”, recorded in the 2009 edition of “Standards of Traditional Chinese Medicines in Hunan Province” and the 2020 edition of “Pharmacopeia of the People’s Republic of China”, and it can suppress cough, relieve wheezing, dispel phlegm, unblock lac feminium, clear heat, and remove toxins (Commission, C. P., 2020). The medicinal use of this plant can be traced back to the Ming Dynasty and was primarily recorded in “Dian Nan Ben Cao” by Mao Lan (1397–1470) (Wang et al., 2018). Extensive evidence indicates that C21 steroid glycosides, extracted by ethyl acetate from TGT, have a significant inhibitory effect against different cancer cell lines, such as A549, Caco-2, SACC83, PC-3, K562, and HepG2 (Ye et al., 2014; Wang et al., 2015). We found that the four C21 steroidal glycosides isolated from TGT had a higher rate of inhibition of A549 cells proliferation than other cell lines (Xu, 2018; Hu et al., 2020). To elucidate the relationship between the chemical structure and cytotoxic activities of steroidal glycosides and to investigate the anti-cancer mechanism of TGT, we isolated and characterized novel compounds from this medicinal plant.

Over the past decade, network-based pharmacological analyses have been employed to assess the mechanisms of herbs and formulae as well as their potential bioactive components at both the molecular and systemic levels (Fang et al., 2017). In particular, network pharmacology has been utilized by Chinese medicine researchers in order to predict the interactions between various components and targets (Mao et al., 2017; Zhang et al., 2019). Furthermore, network pharmacology is also a useful *in silico* prediction tool for identifying active components and elucidating the mechanisms of herbal medicines that, in turn, allows more investigations of these bioactive compounds.

This study developed an approach that integrated cytotoxicity screening, phytochemical analysis, cellular and molecular biology, and network pharmacology construction to identify effective antitumor substances and the underlying mechanisms of TGT. To our knowledge, this is the first integral study that employed several methods to identify efficacious antitumor substances and elucidated their mechanisms of action.

## Materials and Methods

### Chemicals and Materials

The dry cane of TGT was procured from Huayu Pharmaceutical Co., Ltd. (Guangzhou, Guangdong, China). Reference standards of TGT and marsdenoside H (purity >98%) (purity were obtained from the National Institutes for Food and Drug Control (Beijing, China). Four C21 steroidal glycosides, 11*α*-*O*-benzoyl-12*β-O*-tigloyltenacigenin B (TGT-**15**), marsdenoside C (TGT-**7**), 11*α*-*O*-tigloyl-12*β*-*O*-benzoyltenacigenin B (TGT-**9**), and 11*α*-*O*-2-methylbutyryl-l2*β*-*O*-benzoyltenacigenin B (TGT-**13**), were isolated from an ethyl acetate extract of TGT in our laboratory, and their purities were all >98% based on HPLC normalization and silica gel TLC analysis (Xu, 2018). Dulbecco’s modified Eagle’s medium (DMEM) was obtained from Gibco Invitrogen (Carlsbad, CA, United States). Fetal bovine serum (FBS) was obtained from Hyclone, Co. (Fremont, CA, United States). A549 cells were obtained from the National Infrastructure of Cell Line Resource (Beijing, China). Annexin-FITC cell apoptosis assay and cell cycle assay kits were obtained from Sanjian Biotechnology Co. (Tianjin, China). Reactive oxygen species and mitochondrial membrane potential assay kits were obtained from Boster Biological Technology (Wuhan, Hubei, China). Primary antibodies against MMP-2, cleaved caspase-3, Bcl-2, Bax, cytochrome C, and GAPDH were obtained from Abcam (Cambridge, United Kingdom). MMP-9, cleaved caspase-9, and cleaved caspase-8 were obtained from Shanghai Rebiosci Biotechnology Co. (Shanghai, China), and β-actin was obtained from Sigma–Aldrich (St. Louis, MO, United States). A CX-21 Ordinary Optical Microscope was obtained from Olympus (Shanghai, China). A DR-200Bs ELISA instrument was obtained from Wuxi Hiwell Diatek Instruments Co., Ltd (Wuxi, Jiangsu, China). A FACSCalibur flow cytometer was obtained from Becton, Dickinson and Company (BD, United States).

### *Marsdeniae tenacissimae* Extract Preparation

The dry cane of *Marsdeniae tenacissimae* (5.0 kg) was soaked overnight in 85% ethanol-H_2_O until fully saturated and was extracted with 90 l 85% ethanol-H_2_O three times for two hours each time. The ethanol solvent was then concentrated under reduced pressure to yield a crude lysate, of which 940.3 g was obtained using an extractor at room temperature (25°C). The total ethanol extract was dissolved in water with a total volume of 5 l and then partitioned with petroleum ether (4 L × 4) for depigmentation, yielding 35.1 g of extract. The aqueous layer was sequentially partitioned with ethyl acetate (4 L × 8) and n-butanol (4 L × 8) to yield ethyl acetate-soluble (370.1 g) and n-butanol-soluble (340.0 g) fractions, respectively. The remaining part was the aqueous layer (154.7 g).

### *Marsdeniae tenacissimae* Separation and Purification

The ethyl acetate layer extract was dissolved in organic solvent, and thin-layer chromatography was used to screen a solvent elution system. This ethyl acetate-soluble fraction was then subjected to silica gel column chromatography (using 10 times the amount of extract) and was eluted with petroleum ether–acetone (v/v, 50:1, 30:1, 10:1, 5:1, 3:1, 2:1, or 1:1) to obtain fractions Fr.1 to Fr.9 based on their TLC profiles. Fr.3 was mixed with a portion of Fr.4, and Fr.7 was mixed with a portion of Fr.8.

Fraction 1 was fractionated by silica gel column chromatography and was eluted with CHCl_3_–MeOH to obtain six fractions, namely, Fr.1.1–Fr.1.6 based on TLC analysis. Fr.1.3 and Fr.1.5 were fractionated on an ODS column with a 40–80% methanol–water gradient elution to obtain 4 (17.0 mg), 12 (9.6 mg), and 20 (10.5 mg), respectively.

Fraction 2 was fractionated using silica gel column chromatography and was eluted with CHCl_3_–MeOH to obtain six fractions, namely, Fr.2.1–Fr.2.6, based on TLC analysis. Fr.2.1 and Fr.2.4 on the ODS column with a methanol–water gradient were eluted to obtain 3 (17.5 mg), 9 (10.5 mg), and 10 (18.1 mg).

Fraction 3 was separated by silica gel column chromatography and preparative PHPLC (detection at a wavelength of 210 nm, 55% MeOH, 2.2 ml/min) to obtain 1 (10.6 mg), 2 (15.6 mg), 7 (10.8 mg), and 8 (15.6 mg). Fr.3.2 was resolved by ODS column chromatography and was eluted with CHCl_3_–MeOH to obtain 5 (13.3 mg). Fr.3.4 was resolved using ODS column chromatography, eluted with CHCl_3_–MeOH, and assessed using PHPLC (detection wavelength: 210 nm, 30% acetonitrile, and 2.2 ml/min) to obtain 11 (15.6 mg) and 19 (4.2 mg).

Fraction 5 was resolved by silica gel column chromatography and was eluted with CHCl_3_–MeOH to obtain three fractions, Fr.5.1–Fr.5.3, based on TLC analysis. Fr.5.2 was resolved by ODS column chromatography and was eluted with MeOH–water to obtain 6 (4.8 mg).

Fraction 6 was resolved by silica gel column chromatography and was eluted with CHCl_3_–MeOH to obtain four fractions, Fr.6.1–Fr.6.4, based on TLC analysis. Fr.6.1 was resolved by ODS column chromatography and was eluted with MeOH–water to obtain 13 (6.4 mg). Fr.6.4 was also resolved by ODS column chromatography and was eluted with MeOH–water. Subsequently, the distillates were further resolved by Sephadex LH-20 column chromatography and were eluted with MeOH–water to obtain 17 (4.0 mg).

Fraction 7 was resolved by silica gel column chromatography and was eluted with CHCl_3_–MeOH to obtain six fractions, Fr.7.1–Fr.7.6, using TLC analysis. Fr.7.2 was resolved by Sephadex LH-20 column chromatography and was eluted with MeOH–water to obtain 14 (6.4 mg). Fr.7.4 was separated by preparative PHPLC (detection at 210 nm, 30% acetonitrile, 2.2 ml/min) to obtain 15 (4.0 mg).

**FIGURE 1:**
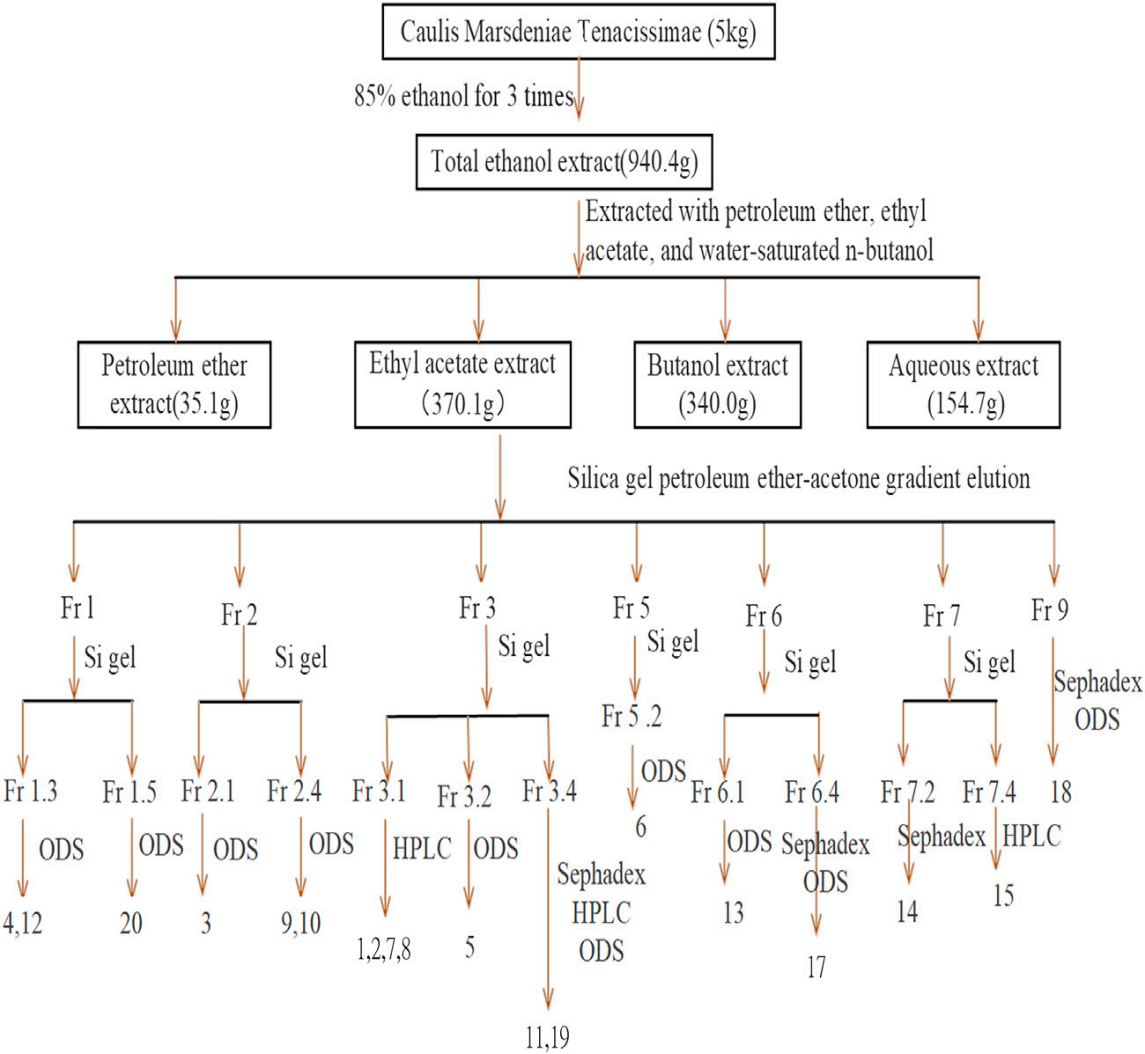
Separation and purification processes.

Fraction 9 was resolved using Sephadex LH-20 column chromatography and was eluted with MeOH–water and was further separated by silica gel column chromatography to obtain 18 (6.0 mg). Figure 1 shows the specific extraction and separation process.

### Spectral Data

Compound 15, white powder,${\left[\alpha \right]}_{D}^{20}$ −43.224 (c 0.0188, CH_3_OH); UV λ_max_ (CH_3_OH)/nm: 202 nm. IR (KBr) λ_max_ 3,384 cm^−1^, 12,963 cm^−1^, 2,930 cm^−1^, 2,868 cm^−1^, 1,736 cm^−1^, 1,456 cm^−1^, 1,364 cm^−1^, 1,170 cm^−1^, 1,070 cm^−1^, 1,022 cm^−1^, 972 cm^−1^, 835 cm^−1^; HR-ESI-MS: m/z 573.4 [M+Na]^+^, 551.6 [M+H]^+^, 585.4 [M+Cl]^−^, 549.1 [M−H]^−^, C_33_H_42_O_7_;^1^H NMR (CDCl_3_, 500 MHz), δ_H_: 1.25 (1H, m, H-1a), 1.25 (1H, m, H-1b), 132 (1H, m, H-2), 3.56 (3H, m, H-3), 1.40 (2H, m, H-3), 1.42 (2H, m, H-4), 2.05 (1H, m, H-6), 1.90 (2H, m, H-7), 2.18 (1H, d, *J* = 10.2 Hz, H-9), 5.66 (1H, t, *J* = 10.2 Hz, H-11, 5.66 (1H, t, *J* = 10.2 Hz, H-125.66 (1H, tH, m, H-15), 2.40 (2H, m, H-16), 2.96 (1H, d, *J* = 7.2 Hz, H-1725.66 (1H, tH, m, H-15)_3_), 1.10 (3H, s, 19-CH_3_), 2.23 (3H, s, 21-CH_3_), 7.88 (2H, d, *J* = 7.8 Hz, H-3′, 7′), 7.36 (2H, t, *J* = 7.8 Hz, H-4′, 6′), 7.50 (1H, t, *J* = 7.2 Hz, H-5′), 7.36 (2H, t, *J* = 7.8 Hz, H-4′, 6′), 7.88 (2H, d, *J* = 7.8 Hz, H-3′,7′), 6.57 (1H, q, *J* = 6.0 Hz, H-3″), 1.49 (3H, d, *J* = 6.6 Hz, 4″-CH3), 1.45 (3H, s, 5″-CH_3_).^13^C NMR (CDCl_3_,125 MHz) δ_C_: 37.3 (C-1), 31.3 (C-2), 70.5 (C-3), 38.3 (C-4), 44.0 (C-5), 26.7 (C-6), 31.8 (C-7), 66.9 (C-8), 51.2 (C-9), 38.9 (C-10), 69.7 (C-11), 74.7 (C-12), 46.1 (C-13), 71.5 (C-14), 26.7 (C-15), 25.0 (C-16), 59.8 (C-17), 16.6 (C-18), 12.8 (C-19), 211.0 (C-20), 30.3 (C-21), 166.1 (C-1′), 130.3 (C-2′), 129.6 (C-3′), 128.2 (C-4′), 132.9 (C-5′), 128.2 (C-6′), 129.6 (C-7′), 167.4 (C-1″), 127.6 (C-2″), 138.3 (C-3″), 14.2 (C-4″), 11.5 (C-5″).

Compound **18**, white powder, ${\left[\alpha \right]}_{D}^{\mathrm{20}}$16.118 (c 0.0225, CH_3_OH); UV λ_max_ (CH_3_OH)/nm: 216 nm; IR λ_max_: 3,390 cm^−1^, 2,927 cm^−1^, 2,860 cm^−1^, 1,687 cm^−1^, 1,441 cm^−1^, 1,382 cm^−1^, 1,272 cm^−1^, 1,245 cm^−1^, 1.129 cm^−1^, 1.023 cm^−1^, 897 cm^−1^, 873 cm^−1^; HR-ESI-MS: m/z 695.6 [M+H]^+^, 671.7 [M−Na]^−^, C_29_H_51_NaO_17_; ^1^H NMR (CD_3_OD, 500 MHz), δ_H_: 2.66 (each 1H, dd, *J* = 4.8, 9.6 Hz, H-2), 2.86 (each 1H, dd, *J* = 4.8, 9.6 Hz, H-2), 3.98 (1H, m, H-3), 3.55 (1H, dd, *J* = 3.6, 8.4 Hz, H-4), 3.63 (1H, m, H-5), 1.19 (3H, d, *J* = 7.2 Hz, H-6), 2.61 (each 1H, dd, *J* = 4.8, 9.6 Hz, H-2′), 2.81 (each 1H, dd, *J* = 4.8, 9.6 Hz, H-2′), 4.05 (1H, m, H-3′), 3.61 (1H, m, H-4′), 3.71 (1H, m, H-5′), 1.23 (3H, d, *J* = 7.8 Hz, H-6′), 3.39 (3H, s, H-OCH_3_), 3.68 (3H, s, H-1′-OCH_3_), 3.41 (3H, s, H-3′-OCH_3_), 4.60 (1H, d, *J* = 9.6 Hz, H-Allo-1), 3.18 (1H, m, H-Allo-2), 3.62 (1H, m, H-Allo-3), 3.35 (1H, m, H-Allo-4), 3.92 (1H, m, H-Allo-5), 1.30 (3H, d, *J* = 7.8 Hz, H-Allo-6), 3.59 (3H, s, H-3-OCH_3_), 4.70 (1H, d, *J* = 10.2 Hz, H-Allo-1′), 3.18 (1H, m, H-Allo-2′), 3.62 (1H, m, H-Allo-3′), 3.35 (1H, m, H-Allo-4′), 4.32 (1H, m, H-Allo-5′), 1.47 (3H, d, *J* = 7.8 Hz, H-Allo-6′), 3.61 (3H, s, H-3′-OCH_3_); ^13^C NMR (CD_3_OD, 125 MHz) δ_C_: 173.4 (C-1), 33.8 (C-2), 79.7 (C-3), 84.0 (C-4), 71.1 (C-1), 18.1 (C-5), 174.2 (C-1′), 36.3 (C-2′), 79.0 (C-3′), 82.6 (C-4′), 71.1 (C-5′), 18.1 (C-6′), 57.5 (C-3-OCH_3_), 52.1 (C-1′-OCH_3_), 58.9 (C-3′-OCH_3_), 102.7 (C-Allo-1), 75 (C-Allo-2), 83.7 (C--Allo-4), 73.6 (C-Allo-4), 68.3 (C-Allo-5), 20.0 (C-Allo-6), 62.5 (C-3-OCH_3_), 103.9 (C-Allo-1′), 74.9 (C-Allo-2′), 83.7 (C-Allo-3′), 74.0 (C-Allo-4′), 78.0 (C-Allo-5′), 19.3 (C-Allo-6′), 62.5 (C-3′-OCH_3_).

### Constituent Identification

All LC-MS and MS/MS results were processed using MassLynx™ (V4.1). Molecular formula estimations of the compounds were performed using Elemental Composition software (Waters Technologies, Milford, MA, United States). Structure determination of the main compounds, such as the chemical structure, precise molecular mass, as well as potential molecular fragmentation pathways, was performed using Mass Fragment software. Previously published compounds were collected by comprehensively searching various databases such as PubMed^1^, Chemspider^2^, HMDB^3^, and Metlin^4^ (Guijas and Siuzdak, 2018). Validation of compounds with standard materials was performed using reference standards.

### Cell Culture

A549 cells were propagated in Dulbecco’s modified Eagle medium (DMEM) supplemented with 10% fetal bovine serum (FBS) and 1% penicillin-streptomycin at 37°C in a humid atmosphere with 5% CO_2_.

### Cytotoxicity Experiment

The cytotoxic effects of *M. tenacissimae* extracts were assessed using the CCK-8 calorimetric procedure. Briefly, cells (density: 5 × 10^3^ cells/well) were seeded into 96-well plates and cultured for 24 h. The supernatant was then discarded, and the cells were treated with extracts at various concentrations using a volume of 100 ul in 96-well plates to which DMEM was added with serum. Five wells were used for each concentration. Five blank controls with only the medium (no addition of cells) were used as the negative control group. After incubation for 24 h, the original medium in each well was replaced with 100 μl of medium containing 10% CCK-8. Thereafter, the 96-well plates were placed in an incubator for 3 h. The absorbance of each well at a wavelength of 450 nm was then determined with a microplate reader (TECAN, Switzerland). The experiment was performed three times in parallel.

### Network Pharmacology Construction and Analysis

ChemSketch was used to draw the structure of the above six C21 steroidal saponins and to obtain their SMILES number. Then, we used SciFinder^5^ to confirm their molecular structure and obtained their CAS numbers. The Swiss Target Prediction^6^ (Gfeller et al., 2014) and STITCH^7^ (Kuhn et al., 2010) databases were used to screen potential targets of six C21 steroidal glycosides. The DigSee (Kim et al., 2017). DisGeNET^8^ (Pinero et al., 2015) and OMIM^9^ databases (Amberger et al., 2015) were employed to screen potential targets of NSCLC. Furthermore, the targets of disease were matched with the targets of the compound, and a compound-target-NSCLC network was constructed with Cytoscape 3.7.2 (Settle et al., 2018). The KEGG^10^ database (Amberger et al., 2015) was used to enrich the target signal pathway. Then, the compound-target and target-pathway networks were merged to obtain a compound-target-pathway network. String APP in Cytoscape 3.7.2 was employed, and the Network analysis and Generatestyle functions were used to generate the protein-target-interaction network. DAVID^11^ was used to conduct Gene Ontology (GO) and KEGG pathway analyses. Finally, we used the KEGG Mapper tool to obtain and integrate the pathway related to the anti-NSCLC effect of the six C21 steroidal saponins.

### Migration Assay

The IC_50_ values of compounds TGT-7, TGT-9, TGT-13, and TGT-15 were determined to be 28.36, 44.01, 29.03, and 47.33 μm, respectively, in a previous study (Xu, 2018; Hu et al., 2020). Based on the IC_50_ values of compounds, we determined the concentration necessary for tests with A549 cells. A549 cells (density: 1 × 10^6^ cells per well) were first seeded into six-well plates. Upon reaching a confluency of 90%, the A549 monolayer was scraped in the middle of each well using a 20 μl pipette tip, and then the plates were washed three times with PBS, and media with TGT-**7**, TGT-**9**, TGT-**13**, and TGT-**15** were added separately at concentrations of 28 μM, 44 μM, 29 μM, and 47 μM, respectively, for 36 h. The control group was supplemented with DMEM. Then, three fields of every wound were selected, and the rate of wound closure was calculated using ImageJ (Wayne Rasband, National Institutes of Health, United States).

## Invasion Assay

Matrigel® was left to stand at 4°C overnight and was then thawed. The Matrigel® gel was prepared with serum-free medium at a final concentration of 1 mg/ml. Approximately 100 μl of the prepared Matrigel® gel was vertically added to the bottom of the upper chamber, and 600 ul of each well consisting of 10% FBS was placed in the lower chamber. Then, 1 × 10^5^ A549 cells were resuspended in 100 µl in the bottom of the well, supplemented with 0.1% BSA and TGT-**7** (28 µM), TGT-**9** (44 µM), TGT-**13** (29 µM), or TGT-**15** (47 µM) and cultured for 48 h to allow cell migration across the filter membrane. Cell fixation was performed with methanol for 30 min, followed by 1% crystal violet staining for 25 min and the washing away of excess crystal violet stain. A total of five images were randomly captured with an inverted microscope, which were then used to count the number of transmembrane cells.

### Cell Cycle Analysis

Cell cycle analysis was conducted using flow cytometry. A549 cells were treated with TGT-**7** (28 µM), TGT-**9** (44 µM), TGT-**13** (29 µM), and TGT-**15** (47 µm) for 24 h, and then the cell pellet was washed three times in ice-cold PBS. The cells were shaken using 70% ethanol and resuspended in a −20°C refrigerator for at least 24 h. After the cells were fixed, they were centrifuged at 1,000 rpm for 5 min. The ethanol was discarded, and the cells were washed three times in ice-cold PBS. The cells were then centrifuged, the supernatant was decanted, and the cell pellet was resuspended in annexin-binding buffer. Then, 0.5 ml of PI/rnase was added to the cells and left to stand in the dark for approximately 15 min. Cell cycle analysis was immediately conducted using flow cytometry.

### Cell Apoptosis Analysis

Cell apoptosis was analyzed using flow cytometry. A549 cells were treated with TGT-**7** (28 μM, 56 µM), TGT-**9** (44 μM, 88 µM), TGT-**13** (29 μM, 58 µM), and TGT-**15** (47 μM, 94 µM) for 24 h. Then, the supernatants were collected in a 15 ml centrifuge tube, and the adherent cells were trypsinized, detached, and collected in a corresponding centrifuge tube and centrifuged at 1,000 rpm for 5 min. Then, the supernatant was discarded, and the cells were washed twice using 1× binding buffer and were sequentially stained with Annexin V and PI following the manufacturer’s instructions. Finally, the A549 cells were observed by fluorescence microscopy and then analyzed by flow cytometry.

### Mitochondrial Membrane Potential Assay

JC-1, a cationic fluorescent dye when added to living cells, is known to be localized exclusively in mitochondria, particularly in good physiological conditions characterized by sufficient mitochondrial membrane potential (∆Ψ). The current paper is dealing with the study of differences in the effects of four compounds (TGT-7, TGT-9, TGT-13, and TGT-15) on the JC-1 loading and fluorescence in A549 cells. A549 cells were treated with TGT-7 (28 µM), TGT-**9** (44 µM), TGT-**13** (29 µM), and TGT-**15** (47 µM), respectively, for 24 h. Then, the A549 cells were stained using JC-1 at 37°C for 20 min, and images were captured using a fluorescence microscope. A decrease in the mitochondrial membrane potential was indicated by a change in the wavelength, i.e., from red to green. ImageJ was used to assess the intensity of red and green fluorescent emissions, which represented potential alterations in the mitochondrial membrane.

### Intracellular ROS Detection

The amount of intracellular ROS produced was determined using an ROS assay. After the A549 cells were treated with TGT-**7** (28 µM), TGT-**9** (44 µM), TGT-**13** (29 µM), and TGT-**15** (47 µM) for 24 h, the A549 cells were incubated in the presence of 10 μM DCFH-DA at 37°C for 30 min, followed by washing twice using PBS. Finally, the A549 cells were assessed under a fluorescence microscope and were processed by flow cytometry to measure DCFH-DA fluorescence.

### Western Blotting

A549 cells that were treated with various concentrations of TGT-**7** (28 and 56 µM), TGT-**9** (44 and 88 µM), TGT-**13** (29 and 58 µM), and TGT-**18** (47 and 94 µM), were lyzed using RIPA buffer containing protease inhibitors. Then, the BCA assay was performed to determine protein concentrations. Proteins were resolved by SDS-PAGE and then immunoblotted onto PVDF membranes. MMP-2, MMP-9, caspase-9, caspase-3, and caspase-8, Bax, Bcl-2, and cytochrome C primary antibodies were used to detect the corresponding proteins, followed by incubation with the corresponding secondary antibodies. Finally, ImageJ (developed by the National Institute of Health) was used to quantify the immunoblots, and the images presented are representative of three separate experiments.

### Statistical Analysis

The experimental data were analyzed using SPSS 20.0. Unless otherwise stated, the data were presented as the arithmetic means of three independent experiments. The results were shown as the mean ± SD. We employed one-way ANOVA to assess variance when homogeneous variance was observed, with the least significant difference (LSD). In addition, the Dunnett T3 test was utilized when the variance was determined to be not uniform. Statistical significance was considered at *p < 0.05, **p < 0.01, and ***p < 0.001 in the analyses of TGT-**7-**, TGT-**9-**, TGT-**13-**, and TGT-**15**-treated *vs.* untreated control cells.

## Results

### Characterization of *Marsdeniae tenacissimae* Caulis Chemical Constituents

**FIGURE 2:**
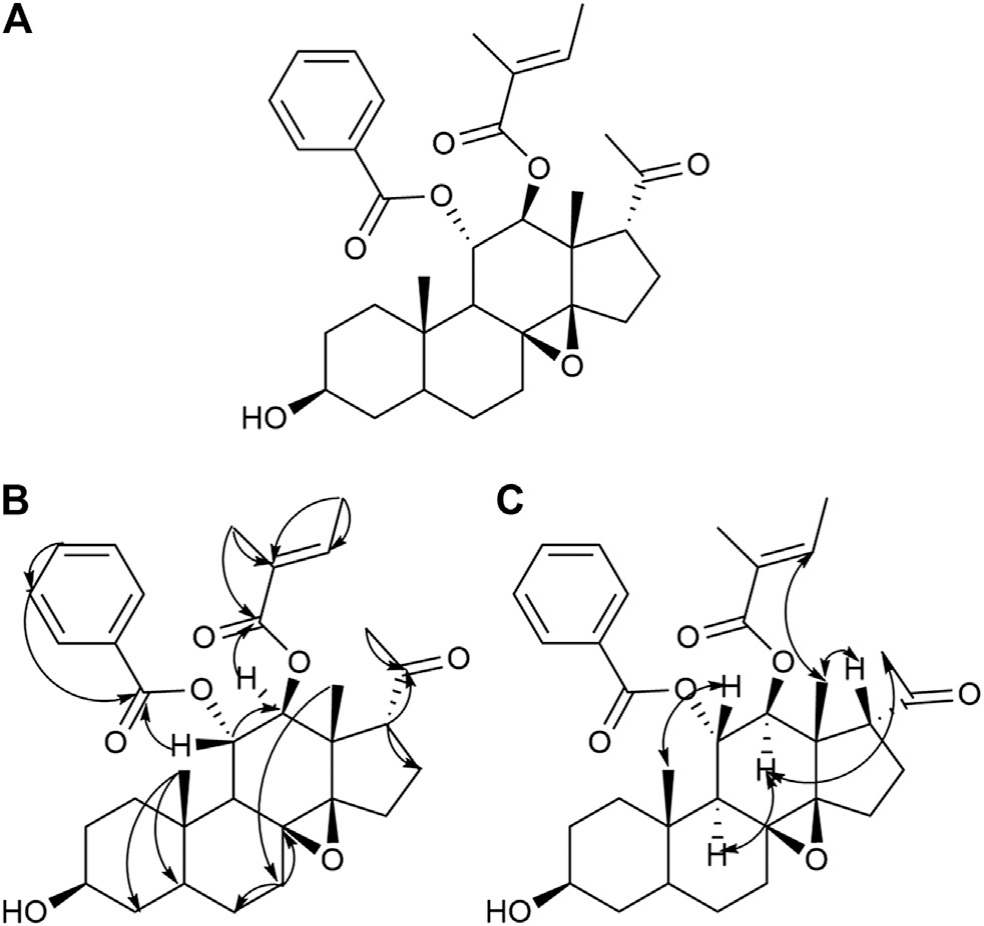
Structure **(A)**, Key HMBC (H→C) correlation **(B),** and Key NOESY (H–H) correlation **(C)** of compound **15**.

**FIGURE 3:**
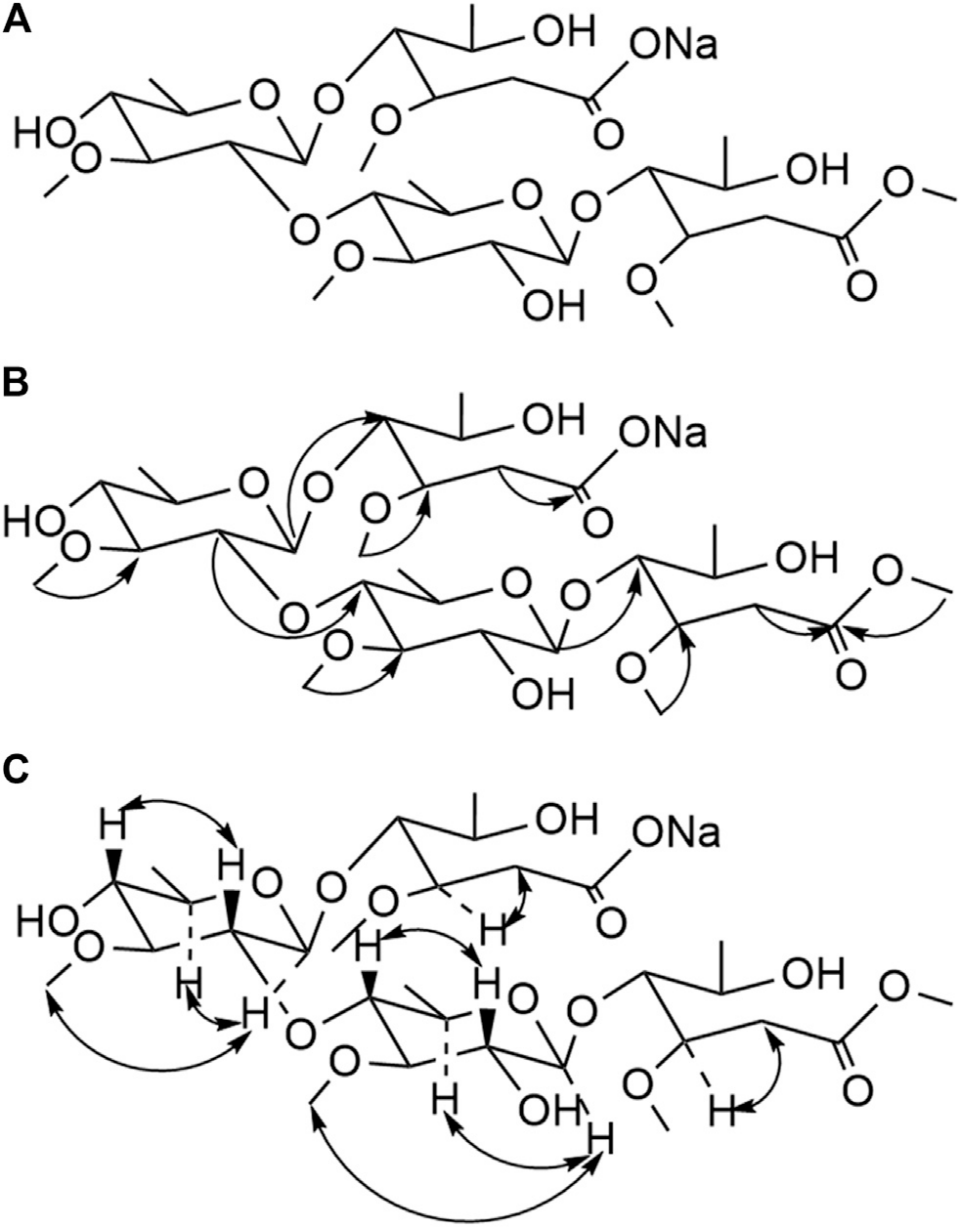
Structure **(A)**, Key HMBC (H→C) correlation **(B),** and Key NOESY (H–H) correlation **(C)** of compound **18**.

**FIGURE 4:**
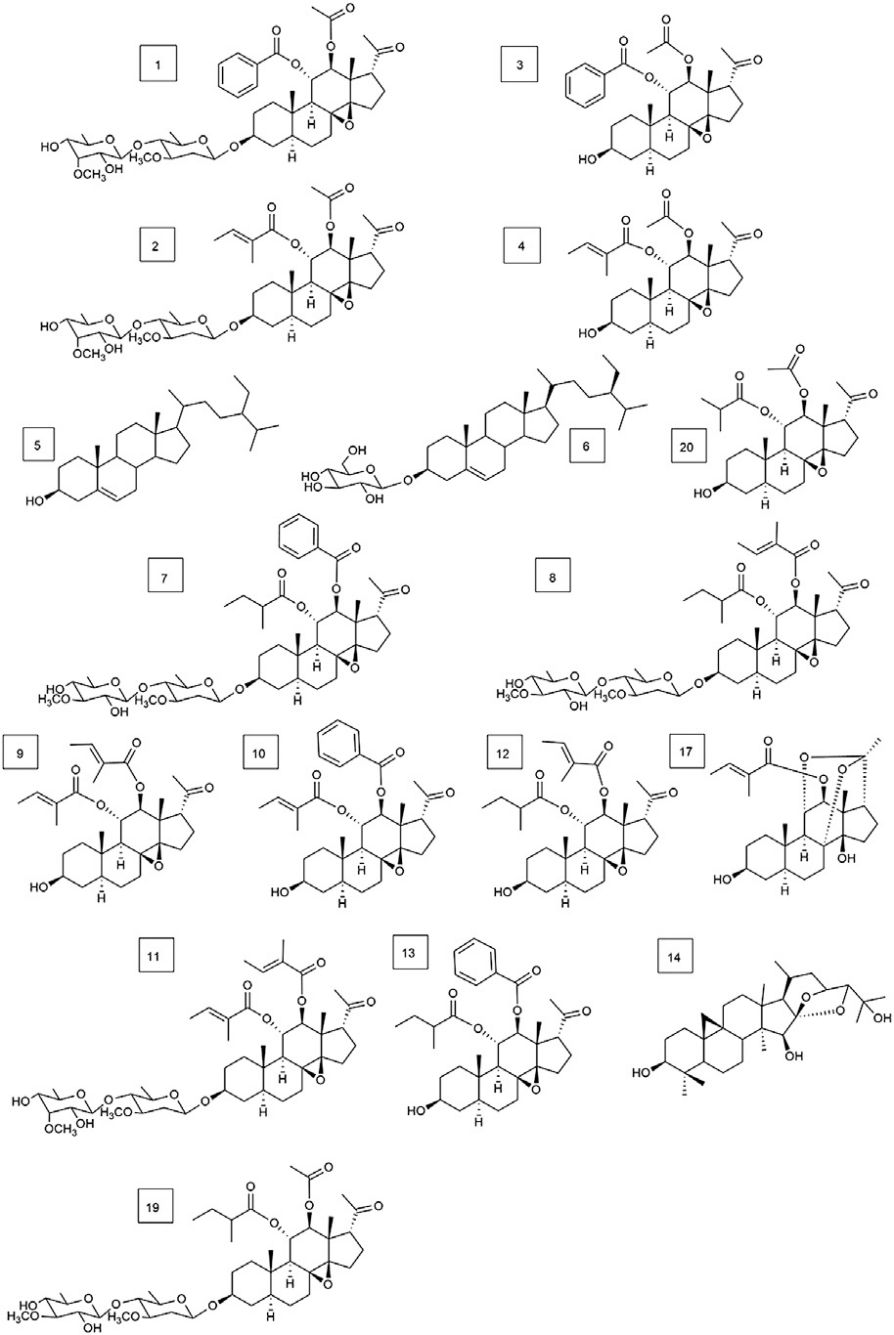
Structures of 17 types of known compounds isolated from *Marsdenia tenacissima* (compounds **1–14**, **17**, **19**, and **20.**).

In this study, we reported the isolation of 19 compounds from the dry cane of *Marsdeniae tenacissimae* Caulis. We elucidated their structures, which included two novel compounds, namely, 11*α*-*O*-benzoyl-12*β*-*O*-tigloyltenac-igenin B (15, 4.0 mg) (Figure 2, Table 1) and sodium 5-hydroxy-4-(((2*S*,3*R*,4*S*,5*R*,6*R*)-5-hydroxy-3-(((2*R*,3*R*,4*R*,5R,6*S*)-5-hydroxy-6-((2-hydroxy-4,6-dimethoxy-6-oxohexan-3-yl)oxy)-4-methoxy-2-methyltetrahydro-2H-pyran-3-yl)oxy)-4-methoxy-6‐methyltetrahydro-2H-pyran-2-yl)oxy)-3-methoxyhexanoate (18, 6.0 mg) (Figure 3, Table 1), as well as 17 other known compounds (1–14, 17, 19, 20) (Figure 4, Supplementary Tables S1, S2).

**TABLE 1:** ^1^H (500 MHz) and ^13^C (150 MHz) NMR date of compounds 15 (in CDCl^3^) and 18 (in MeOD).

Position	Compound 15		Compound 18	
	*δ*_C_ (ppm)	*δ*_H_ (ppm)	*δ*_C_ (ppm)	*δ*_H_ (ppm)
1	37.3	1.25, 1.61, each 1H, m	173.4	
2	31.3	1.32, m	33.8	2.66, 2.86, each 1H, dd, *J* = 4.8, 9.6 Hz
3	70.5	3.56 (1H, m, H-3)	79.7	3.98, 1H, m
4	38.3	1.40, m	84.0	3.55, 1H, dd, *J* = 3.6, 8.4 Hz
5	44.0	1.42, m	71.1	3.63, 1H, m
6	26.7	2.05, m	18.1	1.19, 3H, d, *J* = 7.2 Hz
7	31.8	1.90, m		
8	66.9			
9	51.2	2.18 (1H, d, *J* = 10.2Hz, H-9)		
10	38.9			
11	69.7	5.66(1H, t, *J* = 10.2Hz, H-11β)		
12	74.7	5.17 (1H, d, *J* = 10.2 Hz, H-12α)		
13	46.1			
14	71.5			
15	26.7	2.14, m		
16	25.0	2.40, m		
17	59.8	2.96 (1H, d, *J* = 7.2Hz, H-17β)		
18	16.6	1.13 (3H, s, 18-CH_3_)		
19	12.8	1.10 (3H, s, 19-CH_3_)		
20	211.0			
21	30.3	2.23 (3H, s, 21-CH_3_)		
Bz				
1′	166.1		174.2	
2′	130.3		36.3	2.61, 2.81, each 1H, dd, *J* = 4.8, 9.6 Hz
3′	129.6	7.88 (2H, d, *J* = 7.8Hz, H-3′,7′)	79.0	4.05, 1H, m
4′	128.2	7.36 (2H, t, *J* = 7.8 Hz, H-4′, 6′)	82.6	3.61, 1H, m
5′	132.9	7.50 (1H, t, *J* = 7.2Hz, H-5′)	71.1	3.71, 1H, m
6′	128.2	7.36 (2H, t, *J* = 7.8 Hz, H-4′, 6′)	18.1	1.23, 3H, d, *J* = 7.8 Hz
7′	129.6	7.88 (2H, d, *J* = 7.8Hz, H-3′,7′)		
Tig				
1′′	167.4			
2′′	127.6			
3′′	138.3	6.57 (1H, q, *J*=6.0Hz, H-3″)		
4′′	14.2	1.49 (3H, d, *J*=6.6Hz, 4″-CH_3_)		
5′′	11.5	1.45 (3H, s, 5″-CH_3_)		
4-CH3				
10 (=CH_2_)				
3-O-Me			57.5	3.39, 3H, s
1′-O-Me			52.1	3.68, 3H, s
3′-O-Me			58.9	3.41, 3H, s
Allo-1			102.7	4.60, 1H, d, *J* = 9.6 Hz
2			75	3.18, 1H
3			83.7	3.62, 1H, m
4			73.6	3.35, 1H, m
5			68.3	3.92, 1H, m
6			20.0	1.30, 3H, d, *J* = 7.8 Hz
3-O-Me			62.5	3.59, 3H, s
Allo-1′			103.9	4.70, 1H, d, *J* =10.2 Hz
2′			74.9	3.18, 1H
3′			83.7	3.62, 1H, m
4′			74.0	3.35, 1H, m
5′			78.0	4.32, 1H, m
6′			19.3	1.47, 3H, d, *J* = 7.8 Hz
3′-O-Me			62.5	3.61(3H, s)

Compound **15** was obtained as a white powder. It was identified qualitatively by TLC and colored with anisaldehyde and concentrated sulfuric acid, displaying a yellow–green color, and dark spots were observed under a UV lamp at a wavelength of 254 nm. The ion peaks at m/z 573.4 [M+Na]^+^, 551.6 [M+H]^+^, 585.4 [M+Cl]^–^, and 549.1 [M−H]^–^ for positive and negative ions were obtained using ESI-MS and were assumed to have a molecular weight of 550.3${\left[\alpha \right]}_{D}^{\mathrm{20}}$–43.224 (c 0.0188, CH_3_OH). Elemental analysis indicated that the molecular formula was C_33_H_42_O_7_, and its unsaturation number was 13. The ^1^H-NMR and ^13^C-NMR spectra (Table 1, Supplementary Figure S1) were similar to compound **9** and showed a phenyl ring signal. The ^1^H-NMR spectrum had a monophenyl ring-substituted matrix signal at *δ*_H_ 7.36 (2H, t, *J* = 7.8 Hz), 7.50 (1H, t, *J* = 7.8 Hz), and 7.88 (2H, d, *J* = 7.8 Hz), corresponding to the C (128.2, 129.6, 132.9, 138.3, 166.1) signal on the phenyl ring in our ^13^C-NMR spectrum, and thus was assumed to be a benzoyl moiety. In addition, the ^1^H-NMR spectrum showed a methylbutyryl signal at *δ*_H_ 1.49 (3H, d, *J* = 6.6 Hz, -CH_3_), 1.45 (3H, s, -CH_3_), *δ*_H_ 2.23 (3H, s, 21-CH_3_), and 6.57 (1H, q, *J* = 7.2 Hz), and combined with the ^13^C-NMR spectra at *δ*_C_ 127.6, 130.3 had two olefinic carbon signals, with *δ*_C_ 167.4 being the carbonyl signal of the methacryloyl group. In addition, *δ*_C_ 211.0 was a 20-position carbonyl signal. In the HMBC spectrum (Figure 2B) and Supplementary Figure S1), the 11*β* hydrogen *δ*_H_ 5.66 (1H, t, *J* = 10.2 Hz, H-11*β*) was related to *δ*_C_ 166.1 in benzoyl, showing the benzoyl at the C_11_ position of the aglycone. The 12*α*-hydrogen *δ*_H_ 5.17 (1H, d, *J* = 10.2 Hz, H-12e was related to the carbonyl carbon *δ*_C_167.4 in the methacryloyl group, which indicated that the methacryloyl group was attached to the carbon atom at the 12-position. The 17-site conformation was further confirmed by NOESY spectroscopy (Figure 2C and Supplementary Figure S1). There was an NOE effect between *δ*_H_ 1.13 (H-18) and 2.96 (H-17) in the NOESY spectrum, indicating that the 17-position hydrogen of compound 15 was in the *β*-configuration. A NOE effect was present between *δ*_H_ 1.10 (H-19) and 5.66 (H-11), and the 11-position hydrogen of compound 15 was in the *β*-configuration. Based on the above analysis, compound **15** was determined to be 11*α*-*O*-benzoyl-12*β*-*O*-tigloyltenacigenin B (Figure 2A).

Compound **18** was obtained as a white powder. The ion peaks, m/z 695.6 [M+H]^+^, 671.7 [M−Na]^−^, for the positive and negative ions, respectively, were obtained using ESI-MS and were assumed to have a molecular weight of 694.3${\left[\alpha \right]}_{D}^{\mathrm{20}}$ 16.118 (c 0.0225, CH_3_OH). Elemental analysis indicated that the molecular formula was C_29_H_51_NaO_17_, and the unsaturation number was 4. According to the ^13^C-NMR spectrum (Table 1, Supplementary Figure S2), there were two carbonyl signals, *δ*_C_ 174.2 and 173.4, and there was no olefin carbon signal. According to the degree of unsaturation, we presumed that the compound had two rings. According to the number of oxygens, we presumed that the oligosaccharide chain broke the linked compound, and the compound had four methyl signals in the high field, *δ*_H_1.19 (3H, d, *J* = 7.2 Hz), 1.23 (3H, d, *J* = 7.8 Hz), 1.30 (3H, d, *J* = 7.8 Hz), and 1.47 (3H, d, *J* = 7.8 Hz), based on ^1^H-NMR spectroscopy (Table 1, Supplementary Figure S2), corresponding to the four methyl carbon signals of the carbon spectrum *δ*_C_18.1, 18.1, 19.3, and 20.0 according to the HSQC spectrum; ^1^H-NMR spectral analysis also revealed that there were five -OCH_3_ signals at *δ*_H_ 3.39 (3H, s), 3.41 (3H, s), 3.59 (3H, s), 3.61 (3H, s), and 3.68 (3H, s). The ^1^H-NMR spectrum indicated sugar end group signals at *δ*_H_ 4.70 (1H, d, *J* = 10.2 Hz) and 4.60 (1H, d, *J* = 9.6 Hz), corresponding to the anomeric carbon signals of the carbon spectrum at *δ*_C_ 103.9 and 102.7. A previous study on *Marsdeniae tenacissimae* Caulis showed that an extracted component contained *Marsdenia sinensis* disaccharide (Shi et al., 2007). We presumed from the source route that the compound was likely to be a cleavage product after polymerization of two *Marsdenia sinensis* disaccharides. In combination with HMBC (Figure 3B) and Supplementary Figure S2), *δ*_H_ 4.6 (allo-H-1) was related to *δ*_C_ 84 (C-4), *δ*_H_ 3.18 (allo-H-2) was related to *δ*_C_74 (allo-C-4′), and *δ*_H_ 4.7 (allo-H-1′) was related to *δ*_C_ 82.6 (C-4′). This showed the position of each sugar unit structure of compound **18** in the connection position. A combination of the HSQC, NOESY (Figure 3C), and ^1^H-^1^H-COSY profiles (Supplementary Figure S2) indicated that compound **18** was identified as 2-*Marsdenia sinensis* dimethyl ester-*Marsdenia sinensis* sodium bicarbonate (Figure 3A).

In addition, 17 known compounds (**1–14, 17, 19, 20**) were isolated from the ethyl acetate extract of *Marsdeniae tenacissima* Caulis (Figure 4) and were identified as tenacissoside I (1, 10.6 mg), tenacissoside G (2, 15.6 mg) (Zhang et al., 2010), 11*α*-*O*-benzoyl-12*β*-*O*-acetyltenacigenin B (3, 17.5 mg) (Yao et al., 2014), 11*α*-*O*-tigloyl-12*β*-*O*-acetyltenacigenin B (4, 17.0 mg), *β*-sitosterol (5, 13.3 mg), daucosterol (6, 4.8 mg) (Dong and Cui, 2013), marsdenoside C (7, 10.8 mg), marsdenoside A (8, 15.6 mg) (Deng et al., 2005), 11*α*-*O*-tigloyl-12*β*-*O*-benzoyltenacigenin B (9, 10.5 mg) (Liu and Kong, 2018), 11*α*-*O*-2-methylbutyryl-12*β*-*O*-tiglo-yltenacigenin B (10, 18.1 mg), marsdenoside B (11, 15.6 mg), 11*α*, 12*β*-*O*, *O*-ditigloyl-17*β*-tenacigenin B (**1**2, 9.6 mg) 11*α*-*O*-2-methylbutyryl-l2*β*-*O*-benzoyltenacigenin B (13, 6.4 mg), cimigenol (14, 6.4 mg), 12*β*-*O*-tigloyltenacigenin A (17, 4.0 mg) (Li and Sun, 2008), tenacissoside H (19, 4.2 mg), and 11*α*-*O*-2-methylbutyryl-12*β*-*O-*acetyltenacigenin B (20, 10.5 mg). Cimigenol 14 was the first compound isolated from these extractions. The 1H NMR spectrum and 13C NMR spectrum spectrum data of these compounds are detailed in the supplementary file (Supplementary File S1, Supplementary Tables S1–S3).

### Cell Cytotoxicity Assay of *Marsdeniae tenacissimae* Extracts

**FIGURE 5:**
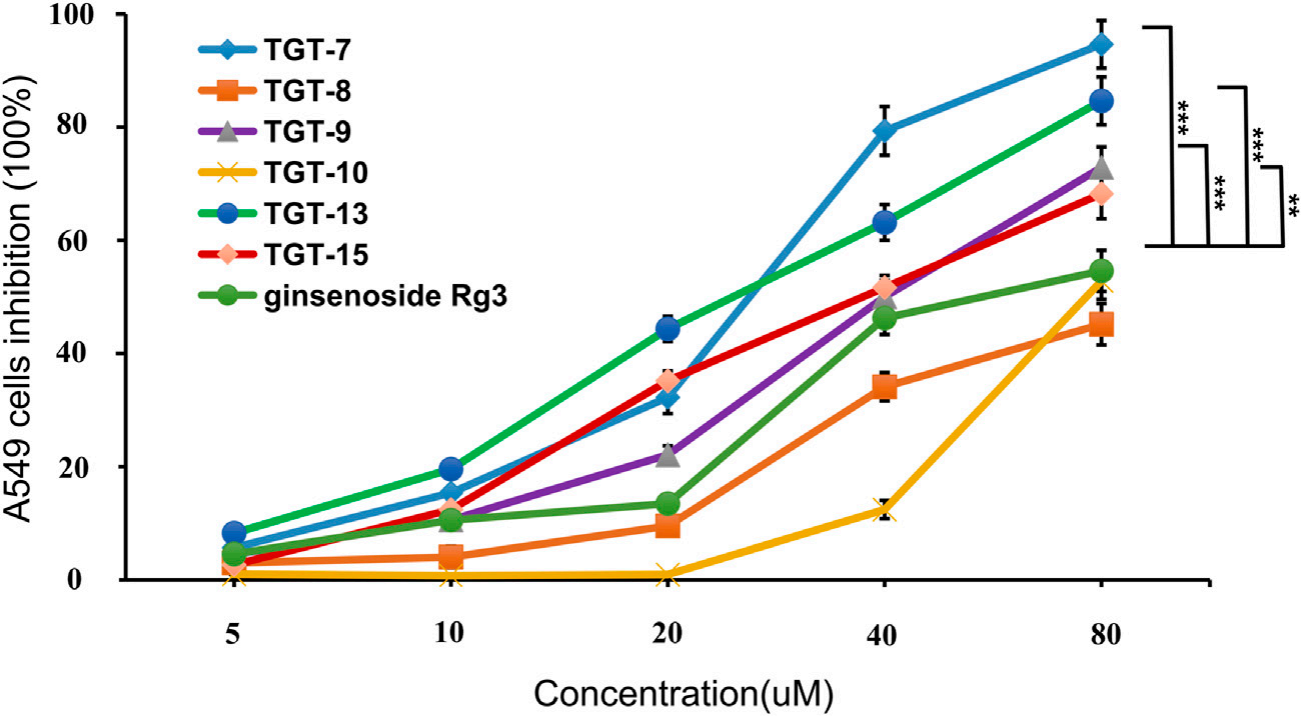
Cytotoxic activity of compounds 7, 8, 9, 10, 13, 15 and ginsenoside Rg3 against the growth of A549 cells. Values are expressed as the mean ± SD,***p < 0.001, **p < 0.01 vs. Ginsenoside Rg3 IC50 (*n* = 3).

The inhibitory activity of all of the isolated compounds against A549 cells was assessed using an *in vitro* assay. The effect of *Marsdeniae tenacissimae* Caulis monomer compounds on the activity of A549 cells showed that six steroidal saponins effectively had inhibitory effects on A549 cells *in vitro*. The IC_50_ values of these six compounds and ginsenoside Rg3 were compared and were arranged in decreasing order as follows: compound **7** < compound **13** < compound **9** < compound **15** < ginsenoside Rg3 < compound **10** < compound **8** (Figure 5, Table 2).

**TABLE 2:** The inhibitory effect of 19 compounds from *Marsdeniae tenacissimae* and Ginsenoside Rg3 on A549 cells.

Compound	IC_50_, µm	Compound	IC_50_, µm
TGT 1-6	/	TGT-12	/
TGT-7	28.36 ± 1.96***	TGT-13	29.03 ± 2.05***
TGT -8	78.14 ± 1.85	TGT-14	/
TGT -9	44.01 ± 1.74***	TGT-15	47.33 ± 2.23**
TGT -10	77.47 ± 2.38	TGT-17-20	/
TGT -11	99.80 ± 2.13	Ginsenoside Rg3	57.32 ± 2.03

Tip: “/” represents the compound IC_50_ > 100 µm,***P < 0.001, **P < 0.01 vs.Ginsenoside Rg3 IC_50_ (*n* = 3).

### Prediction of Putative Targets of *Marsdeniae tenacissimae*

#### Chemical Information and Construction of the Compound-Target-NSCLC Network

SMILES numbers of the compounds were downloaded from the Swiss Target Prediction (Daina et al., 2019) and STITCH databases (Kuhn et al., 2010) used in screening potential targets of the six effective C21 steroidal glycosides. A total of 247 potential targets of the six steroidal saponins were predicted (37 for TGT-**7**, 58 for TGT-**8**, 1 for TGT-**9**, 149 for TGT-**10**, 1 for TGT-**13**, and 1 for TGT-**15**) using the Swiss Target Prediction and STITCH databases, and 205 targets remained after deleting duplicates and false positives.

Searching for NSCLC targets was performed using DigSee (275, Evidence Sentence Score ≥0.6), DisGeNET (225, Score ≥0.1), Malacards (62), and OMIM (142). After the removal of overlapping genes, 270 NSCLC-related targets remained.

**FIGURE 6:**
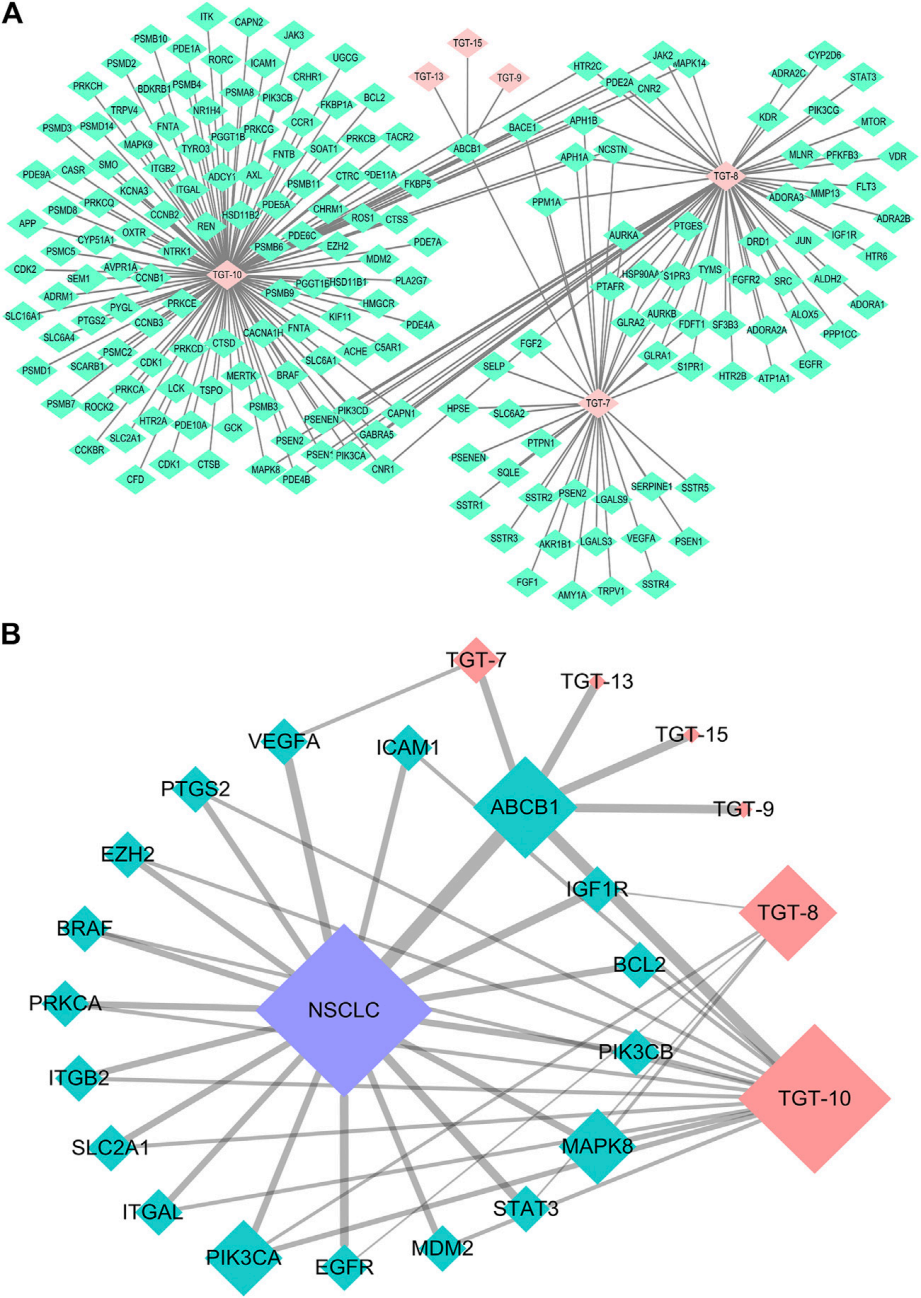
Network pharmacology approach of compounds 7, 8, 9, 10, 13, and 15 **(A)** Compound-target network. Pink represents C21 steroidal saponins (TGT-7, TGT-8, TGT-9, TGT-10, TGT-13, and TGT-15), and green represents their respective targets **(B)** Component-target-NSCLC network. Pink represents C21 steroidal saponins, blue represents NSCLC disease, and green represents their common targets.

Finally, 18 distinct potentially therapeutic genes were identified as targets of the six C21 steroidal glycosides components. This network showed that in terms of anti-NSCLC activity, multiple components acting on multiple targets acted synergistically. Basic information on the six C21 steroidal saponins of *Marsdeniae tenacissimae* Caulis is shown in Table 3 and Figure 6.

**TABLE 3:** 1 Basic information on the six C21 steroidal saponins.

Number	Compound	Molecular formula	Molecular mass (g/moL)	CAS	Degree	Bioavailability	Betweenness centrality
TGT-7	Marsdenoside C	C_47_H_68_O_14_	857.03	858360-58-4	37	0.17	0.202702
TGT-8	Marsdenoside A	C_45_H_70_O_14_	835.03	858360-56-2	58	0.17	0.321595
TGT-9	11α-O-Tigloyl-12β-O-benzoyltenacigenin B	C_33_H_42_O_7_	550.68	1854092-75-3	1	0.55	0
TGT-10	11α-O-2-Methylbutyryl-12β-O-tigloyltenacigenin B	C_31_H_46_O_7_	530.69	154022-54-5	149	0.55	0.857652
TGT-13	11-α-O-2-Methylbutyryl-l2β-O-benzoyltenacigenin B	C_33_H_44_O_7_	552.7	154022-55-6	1	0.55	0
TGT-15	11α-O-Benzoyl-12β-O-tigloyltenacigenin B	C_33_H_42_O_7_	550.68	2288756-09-0	1	0.55	0

#### Construction of the Compound-Target-Pathway Network

The KEGG and DAVID databases were then employed to enrich the target signal pathways. The network of the compound-target-pathway was constructed by Cyctoscape 3.7.2, consisting of 44 nodes and 175 edges. The edges indicated the interactions between active ingredients and targets and pathways.

**FIGURE 7:**
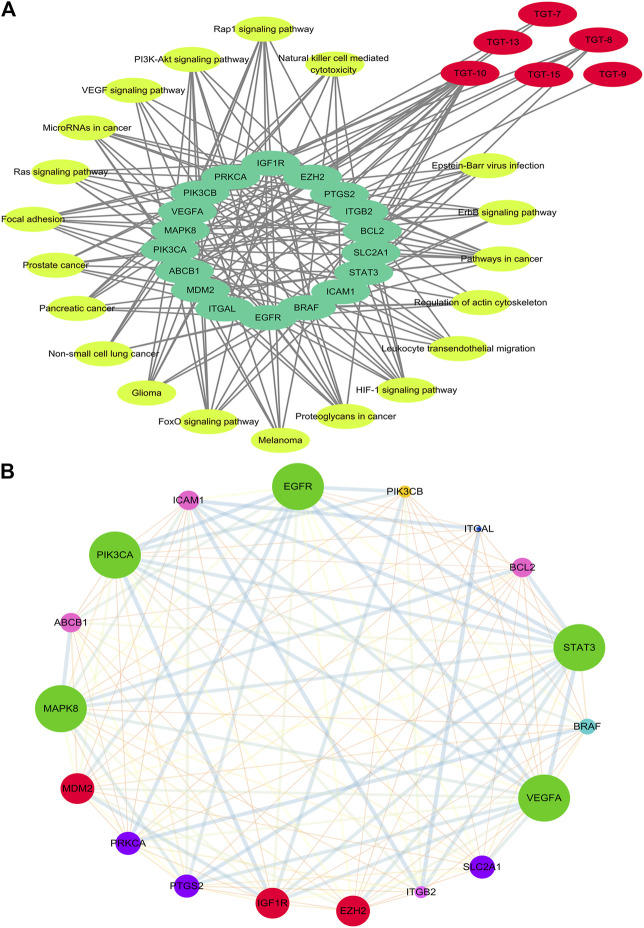
Network of Compound-target-pathway and Protein–protein interaction **(A)** Compound-target-pathway network. Red represents C21 steroidal saponins, bright yellow represents pathways, and green represents their targets **(B)** Protein–protein interaction network (PPI). The larger the node, the greater position occupied in the whole network. The lines between nodes represent the interactions between two proteins that are interconnected. Different-colored lines represent various types of interactions. The thicker the line, the closer the interaction.

**FIGURE 8:**
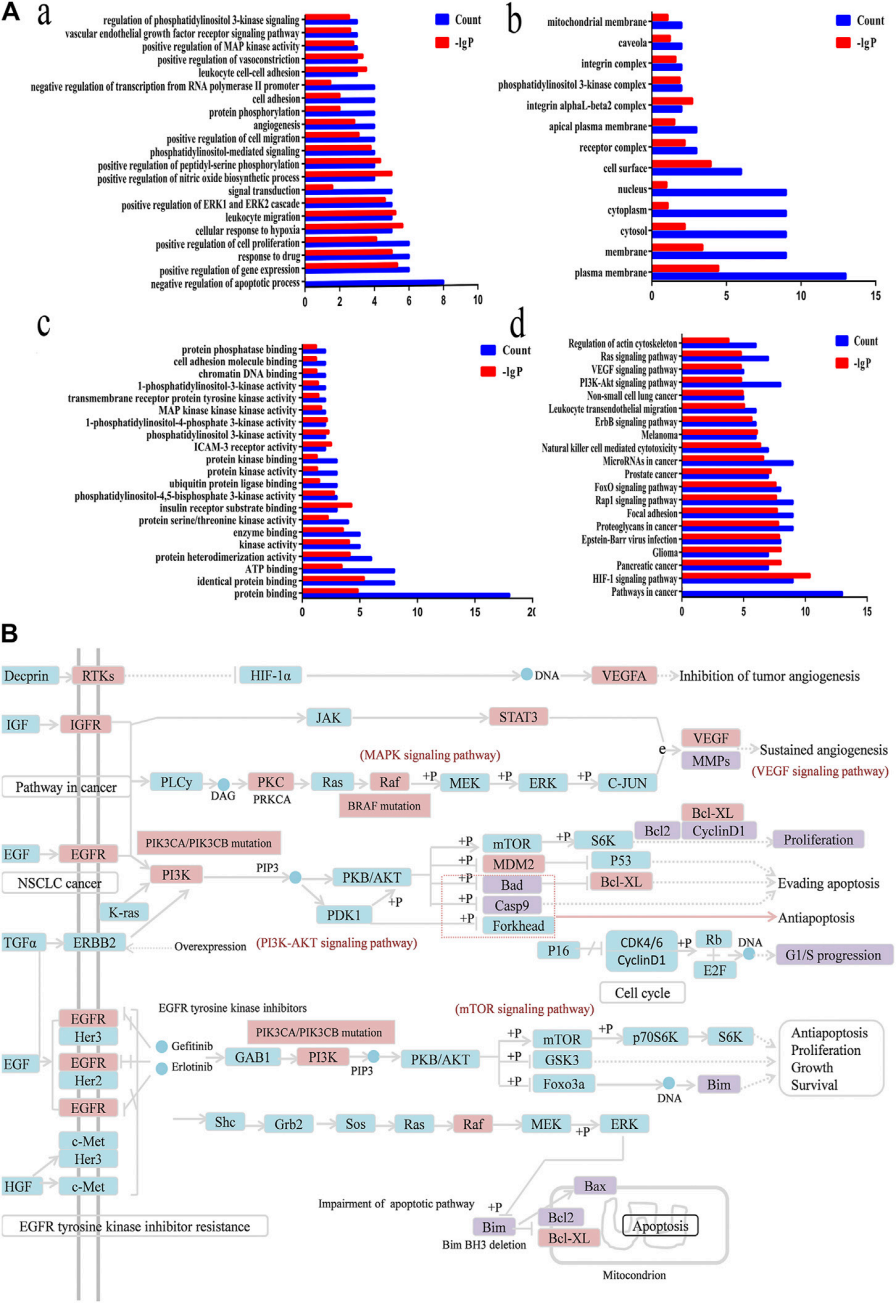
Functional annotation, pathway analysis and Anti-NSCLC pathway of C21 steroidal saponins. **(A)** Functional annotation and pathway analysis of key target genes of C21 steroidal saponins against NSCLC. (a) Enriched Gene Ontology terms for the BP of potential anti-NSCLC targets. (b) MF enrichment analysis of potential anti-NSCLC targets. (c) CC enrichment analysis of potential anti-NSCLC targets. (d) KEGG pathway of anti-NSCLC targets. **(B)** Anti-NSCLC pathway of C21 steroidal saponins. Blue squares represent targets in the pathway, pink squares represent anti-NSCLC targets, and purple squares represent targets that were verified in subsequent experiments in this study.

This network showed that the six C21 steroidal saponins participated in the regulation of different pathways that were related to tumor pathogenesis *via* multi-target synergistic activity. These pathways included cancer-related pathways, PI3K/AKT, HIF-1 pathogenesis *via* multi-target synergistic activity-regulated changes in the tumor cell cycle, and angiogenesis, thus inhibiting cancer cell invasion and migration and inducing tumor apoptosis (Tables 4, 5 Figures 7A, 8A).

**TABLE 4:** Basic information on potential anti-NSCLC targets of C21 steroidal saponins.

Gene	UniProt	Degree	Betweenness centrality	Compound
ABCB1	P08183	6	0.14573713	TGT-7, 9, 10, 13, 15
BCL-2	P10415	8	0.02058895	TGT-10
BRAF	P15056	14	0.04937426	TGT-10
EGFR	P00533	17	0.07256819	TGT-8
EZH2	Q15910	2	0.00068448	TGT-10
IGF1R	P08069	12	0.02322063	TGT-8
ITGAL	P20701	6	0.01079787	TGT-10
ICAM1	P05362	4	0.00486475	TGT-10
ITGB2	P05107	5	0.00718665	TGT-10
MAPK8	P45983	9	0.02437828	TGT-8, 10
MDM2	Q00987	10	0.03250911	TGT-10
PIK3CA	P42336	21	0.12547087	TGT-8, 10
PIK3CB	P42338	20	0.10824658	TGT-10
PRKCA	P17252	15	0.07196801	TGT-10
PTGS2	P35354	4	0.00565462	TGT-10
SLC2A1	P11166	3	0.00236935	TGT-10
STAT3	P40763	8	0.01133759	TGT-8
VEGFA	P15692	11	0.06155873	TGT-7

**TABLE 5:** Key targets and topological properties of C21 steroidal saponins anti-NSCLC.

Gene	Full name	Protein class	Degree	Betweenness centrality	Closeness centrality
EGFR	Epidermal growth factor receptor	None	15	0.15757761	0.89473684
STAT3	Signal transducer and activator of transcription 3	Nucleic acid-binding; transcription factor	13	0.10224673	0.80952381
VEGFA	Vascular endothelial growth factor A	Signaling molecule	13	0.08755544	0.80952381
MAPK8	Mitogen-activated protein kinase 8	kinase; transferase	11	0.0569707	0.73913043
PIK3CA	Phosphatidylinositol-4,5-bisphosphate 3-kinase catalytic subunit alpha	Kinase; transferase	10	0.04892624	0.70833333
PTGS2	Prostaglandin-endoperoxide synthase 2	Oxidoreductase	10	0.0194707	0.70833333
IGF1R	Insulin-like growth factor 1 receptor	None	9	0.02212885	0.65384615
MDM2	MDM2 proto-oncogene	Nucleic acid-binding	8	0.0035014	0.62962963
ITGB2	Integrin subunit beta 2	Cell adhesion molecule; extracellular matrix	7	0.06950572	0.62962963
ICAM1	Intercellular adhesion molecule 1	None	7	0.06517565	0.62962963
SLC2A1	Solute carrier family 2 member 1	None	7	0.00122549	0.60714286
ABCB1	ATP binding cassette subfamily B member 1	Hydrolase; protease	6	0	0.5862069
EZH2	Enhancer of zeste 2 polycomb repressive complex 2 subunit	None	6	0.00105042	0.5862069
PRKCA	Protein kinase C alpha	Calcium-binding protein; kinase; transfer/carrier protein; transferase	5	0.025	0.5862069
BRAF	B-Raf proto-oncogene, serine/threonine kinase	None	4	0.00183824	0.5483871
PIK3CB	Phosphatidylinositol-4,5-bisphosphate 3-kinase catalytic subunit beta	Kinase; transferase	4	0.5483871	0.53846154
BCL-2	BCL-2, apoptosis regulator	Signaling molecule	3	0.00428922	0.51515152

#### Construction and Analysis of the Protein-Interaction Network

STRING was used to assess target protein interactions. Figure 7B shows that the network graph consisted of 18 nodes and 127 edges. The definitions and equations for these parameters revealed the topological significance of the nodes in these networks, and the more important nodes showed higher quantitative values. DisGeNET was used to obtain the corresponding types of targets (Table 6). The results showed that signaling molecules, enzymes, and proteins were involved in the anti-lung cancer effect of *Marsdeniae tenacissima.*

**TABLE 6:** KEEG pathway enrichment of C21 steroidal saponins anti-NSCLC.

Pathway	Count	*P* Value	Protein
Pathways in cancer	13	0.000000	BRAF,BCL2,MDM2,EGFR,IGF1R,MAPK8,PIK3CA,PIK3CB,PTGS2,PRKCA,STAT3,SLC2A1,VEGFA
HIF-1 signaling pathway	9	0.000000	BCL2,EGFR,IGF1R,PIK3CA,PIK3CB,PRKCA,STAT3,SLC2A1,VEGFA
Pancreatic cancer	7	0.000000	BRAF,EGFR,MAPK8,PIK3CA,PIK3CB,STAT3,VEGFA
Glioma	7	0.000000	BRAF,MDM2,EGFR,IGF1R,PIK3CA,PIK3CB,PRKCA
Epstein-Barr virus infection	8	0.000000	BCL2,MDM2,ITGAL,ICAM1,MAPK8,PIK3CA,PIK3CB,STAT3
Proteoglycans in cancer	9	0.000000	BRAF,MDM2,EGFR,IGF1R,PIK3CA,PIK3CB,PRKCA,STAT3,VEGFA
Focal adhesion	9	0.000000	BRAF,BCL2,EGFR,IGF1R,MAPK8,PIK3CA,PIK3CB,PRKCA,VEGFA
Rap1 signaling pathway	9	0.000000	BRAF,EGFR,IGF1R,ITGAL,ITGB2,PIK3CA,PIK3CB,PRKCA,VEGFA
FoxO signaling pathway	8	0.000000	BRAF,MDM2,EGFR,IGF1R,MAPK8,PIK3CA,PIK3CB,STAT3
Prostate cancer	7	0.000000	BRAF,BCL2,MDM2,EGFR,IGF1R,PIK3CA,PIK3CB
MicroRNAs in cancer	9	0.000000	ABCB1,BCL2,MDM2,EZH2,EGFR,PTGS2,PRKCA,STAT3,VEGFA
Natural killer cell mediated cytotoxicity	7	0.000000	BRAF,ITGAL,ITGB2,ICAM1,PIK3CA,PIK3CB,PRKCA
Melanoma	6	0.000001	BRAF,MDM2,EGFR,IGF1R,PIK3CA,PIK3CB
ErbB signaling pathway	6	0.000002	BRAF,EGFR,MAPK8,PIK3CA,PIK3CB,PRKCA
Leukocyte transendothelial migration	6	0.000009	ITGAL,ITGB2,ICAM1,PIK3CA,PIK3CB,PRKCA
Non-small cell lung cancer	5	0.000011	BRAF,EGFR,PIK3CA,PIK3CB,PRKCA
PI3K-Akt signaling pathway	8	0.000015	BCL2,MDM2,EGFR,IGF1R,PIK3CA,PIK3CB,PRKCA,VEGFA
VEGF signaling pathway	5	0.000016	PIK3CA,PIK3CB,PTGS2,PRKCA,VEGFA
Ras signaling pathway	7	0.000016	EGFR,IGF1R,MAPK8,PIK3CA,PIK3CB,PRKCA,VEGFA
Regulation of actin cytoskeleton	6	0.000157	BRAF,EGFR,ITGAL,ITGB2,PIK3CA,PIK3CB

#### Gene Function and Pathway Analysis

DAVID was used to conduct Gene Ontology (GO) and KEGG pathway analyses. A threshold of *p < 0.05* was used in screening the biological process or pathway, and GraphPad Prism 7.0 was employed for drawing enriched terms in the CC, BP, and MF categories (Figure 8A).

BP analysis indicated that these targets were mainly related to biological processes, including negative regulation of apoptotic processes, positive control of cell proliferation, positive control of cell migration, angiogenesis, regulation of phosphatidylinositol 3-kinase signaling, and the vascular endothelial growth factor receptor signaling pathway (Figure 8A).

CC analysis revealed that markedly enriched terms were mainly concentrated in the formation of the phosphatidylinositol 3-kinase complex, plasma membrane, and receptor complex (Figure 8A).

MF analysis showed enriched terms including protein binding, ATP binding, protein serine/threonine kinase activity, phosphatidylinositol-4,5-bisphosphate3-kinase activity, ubiquitin protein ligase binding, phosphatidylinositol3-kinase activity, and 1-phosphatidylinositol-4-phosphate 3-kinase activity (Figure 8A).

#### Target Pathway Analysis

KEGG Mapper was used to obtain the pathway map of *Marsdenia tenacissima* resistance to NSCLC, and the major pathways were integrated to construct a pathway map (Figure 8B). The arrows in the figure indicated promoting effects, T-arrows represented inhibitory effects, the pathway targets were in blue, the network pharmacological prediction targets of resistance to NSCLC were in pink, and the experimentally verified targets were in purple. The figure showed that the anti-NSCLC effects of the six C21 steroidal saponins mainly involved pathways in cancer, including HIF-1 signaling, PI3K-Akt signaling, VEGF signaling, EGFR tyrosine kinase inhibitor resistance, and Ras signaling. The integrated pathway diagram was shown in (Figure 8B). To further explore the specific role and mechanism of its anti-NSCLC, we conducted preliminary experimental verification on the predicted potentially key targets in the pathway.

### Effects of TGT-7, TGT-9, TGT-13, and TGT-15 on the Migration and Invasion of A549 Cells

**FIGURE 9:**
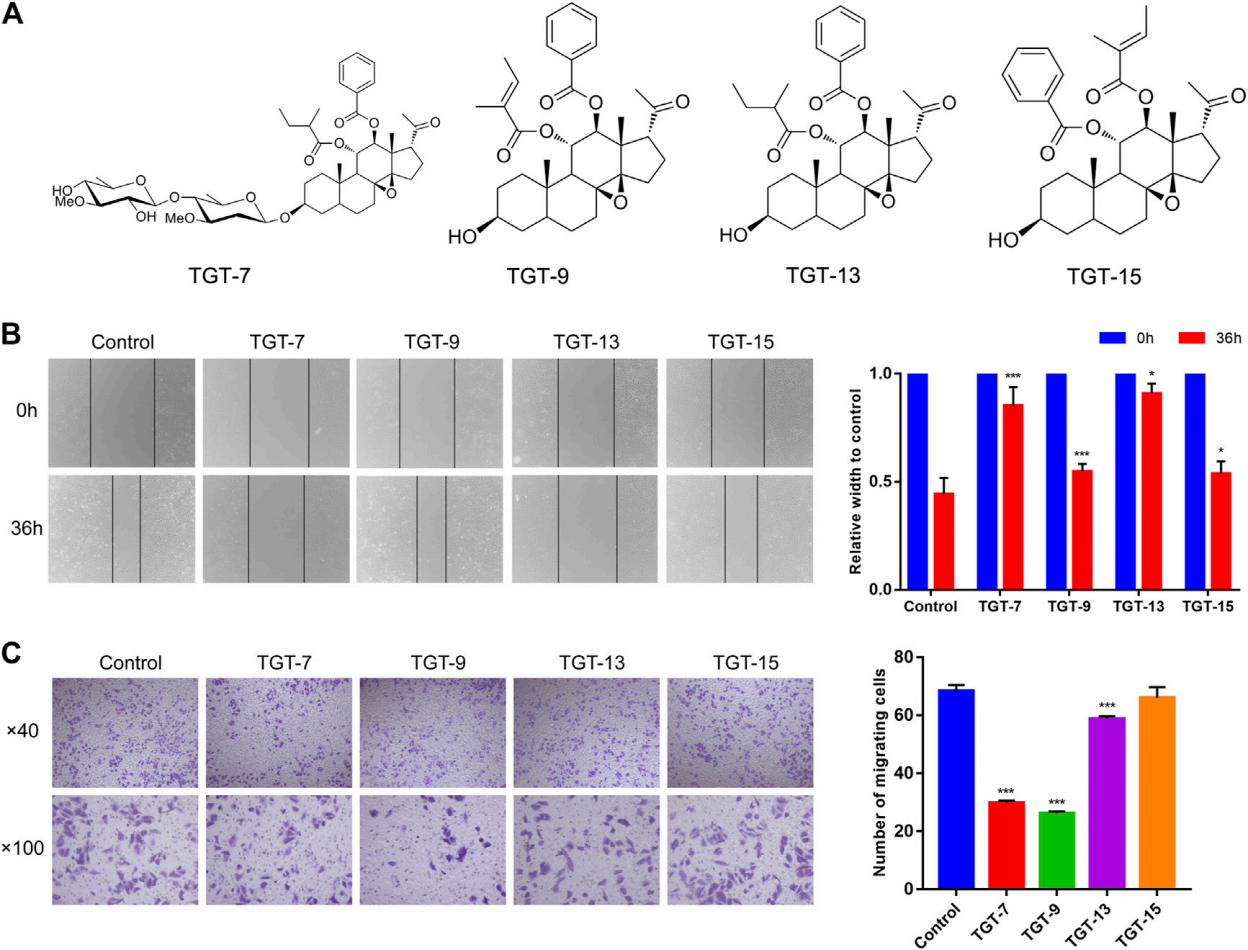
Effects of TGT-7 (28 µm), TGT-9 (44 µm), TGT-13 (29 µm), and TGT-15 (47 µm) on migration and invasion of A549 cells **(A)** Structure of marsdenoside C (TGT-**7**), 11α-*O*-tigloyl-12of*O*-benzoyltenacigenin B (TGT-**9**), and 11-α-*O*-2-methylbutyryl-l2 B*O*-benzoyltenacigenin B (TGT-**13**), 11α-*O*-benzoyl-12ac*O*-tigloyltenacigenin B (TGT-**15**) **(B)** Wound-healing assays were performed to measure the effect of the four C21 steroidal glycosides on the migration ability of A549 cells (original magnification ×40) **(C)** Transwell–Matrigel invasion assay was performed to evaluate the effect of the change in the four C21 steroidal glycosides on the migration ability of A549 cells (original magnification ×100). These results were obtained from three independent experiments, and all of the data are expressed as the mean ± SD, *p < 0.05, ***p < 0.001 vs. the control group (*n* = 3).

To investigate whether TGT-**7**, TGT-**9**, TGT-**13**, and TGT-**15** (Figure 9A) affected the migration and invasion of A549 cells, we first tested the four compounds (TGT-**7**, TGT-**9**, TGT-**13**, and TGT-**15**) in migration and invasion assays. A549 cells were exposed to TGT-**7** (28 µm), TGT-**9** (44 µm), TGT-**13** (29 µm), and TGT-**15** (47 µm) for 36 h. Migration experiments indicated that control cells significantly migrated after treatment with TGT-**7** (28 µm), TGT-**9** (44 µm), TGT-**13** (29 µm), and TGT-**15** (47 µm), with relative widths of the cell scratches of 0.852 ± 0.087, 0.549 ± 0.033, 0.909 ± 0.045, and 0.538 ± 0.056, respectively. Compared with the control group (0.443 ± 0.075), the results were statistically significant (Figure 9B). The invasion assays showed that many A549 cells in the control group were filtered from the upper region of transwell chambers and moved to the lower part after treatment with TGT-**7** (28 µm), TGT-**9** (44 µm), as well as TGT-**13** (29 µm). The number of A549 cells that moved across the filtration membrane significantly decreased. However, after treatment with TGT-**15** (47 µm), the number of A549 cells that moved across the filtration membrane significantly decreased. However, *t*-test results indicated the change was not significant. The number of A549-invading cells after treatment with TGT-7 (28 µm), TGT-9 (44 µm), TGT-13 (29 µm), and TGT-15 (47 µm) were 29.87 ± 0.70, 26.33 ± 0.50, 58.8 ± 0.92, and 66.00 ± 3.74, respectively. Compared with the control group (68.40 ± 2.09), the TGT-**7** (28 µm), TGT-**9** (44 µm), and TGT-**13** (29 µm) groups were statistically significant (Figure 9C). These results showed that TGT-**7**, TGT-**9,** TGT-**13**, and TGT-**15** could inhibit A549 cells migration and invasion.

### TGT-7, TGT-9, TGT-13, and TGT-15 Induced A549 Cells Cycle Arrest at the G0/G1 Phase

**FIGURE 10:**
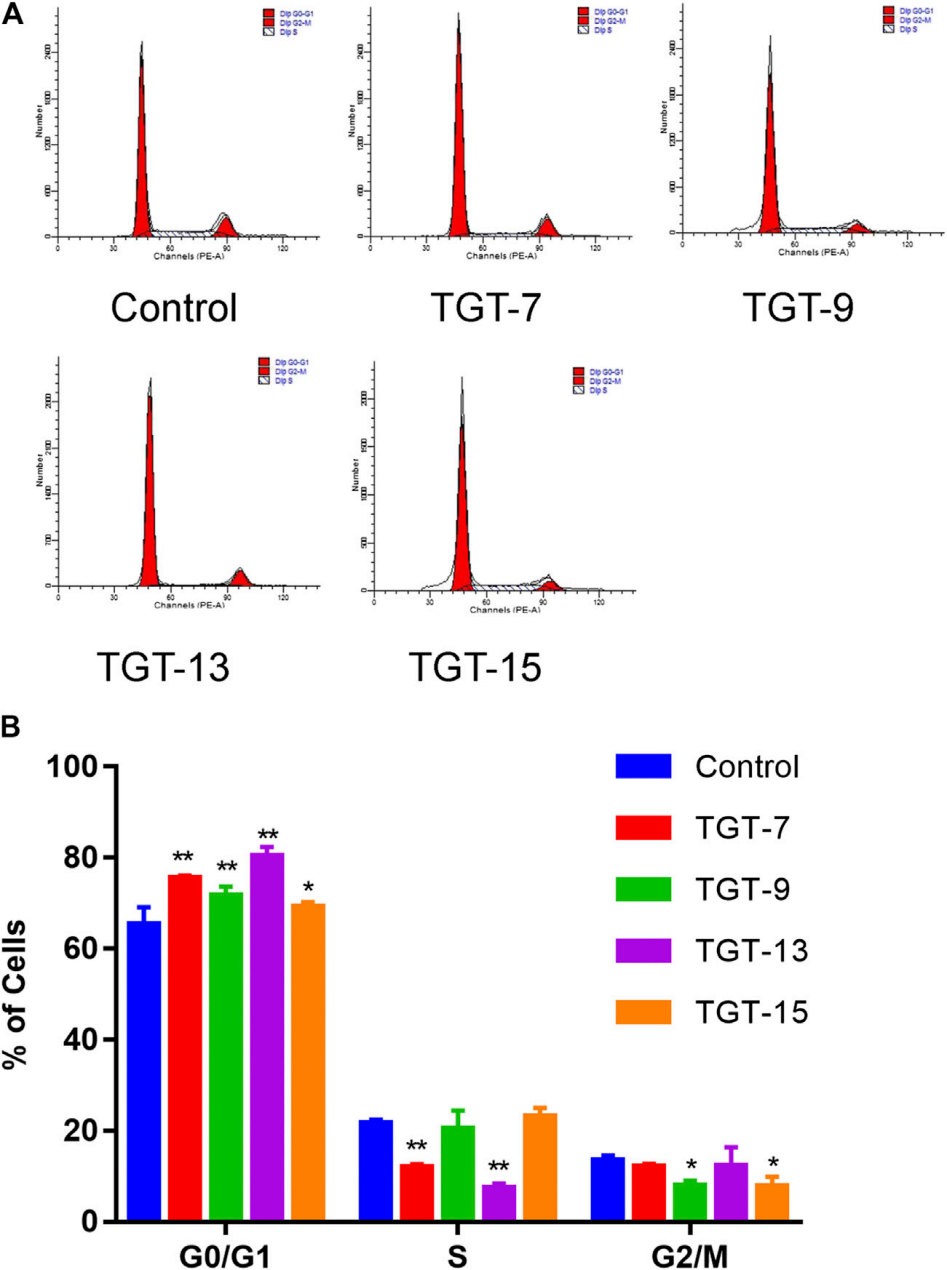
TGT-7 (28 µm), TGT-9 (44 µm), TGT-13 (29 µm) and TGT-15 (47 µm) induce A549 cells cycle arrest in the G0/G1 phase **(A)** Changes in cell cycle phases of A549 cells were detected by flow cytometry **(B)** The number of A549 cells in the G0/G1 phase were significantly increased. These results were obtained from three independent experiments, and all of the data are expressed as the mean ± SD, *p < 0.05, **p < 0.01 vs. the control group (*n* = 3).

A549 cells were exposed to TGT-**7** (28 µm), TGT-**9** (44 µm), TGT-**13** (29 µm), as well as TGT-**15** (47 µm) for 24 h and were fixed and stained using propidium iodide, and alterations in the cell cycle were evaluated by flow cytometry. The results showed that the proportion of G0/G1 cells after treatment with TGT-7 (28 µm), TGT-9 (44 µm), TGT-13 (29 µm), as well as TGT-15 (47 µm) increased from 65.31 ± 3.79% to 75.58 ± 0.44%, 71.63 ± 2.02%, 80.27 ± 2.13%, and 69.17 ± 1.05%, respectively. Compared with the control group, the number of G0/G1 cells increased to different degrees (Figures 10A,B). These results demonstrated that TGT-**7**, TGT-**9**, TGT-**13,** and TGT-**15** could arrest A549 cells at the G0/G1 phase.

### TGT-7, TGT-9, TGT-13, and TGT-15 Induce A549 CellS Apoptosis

**FIGURE 11:**
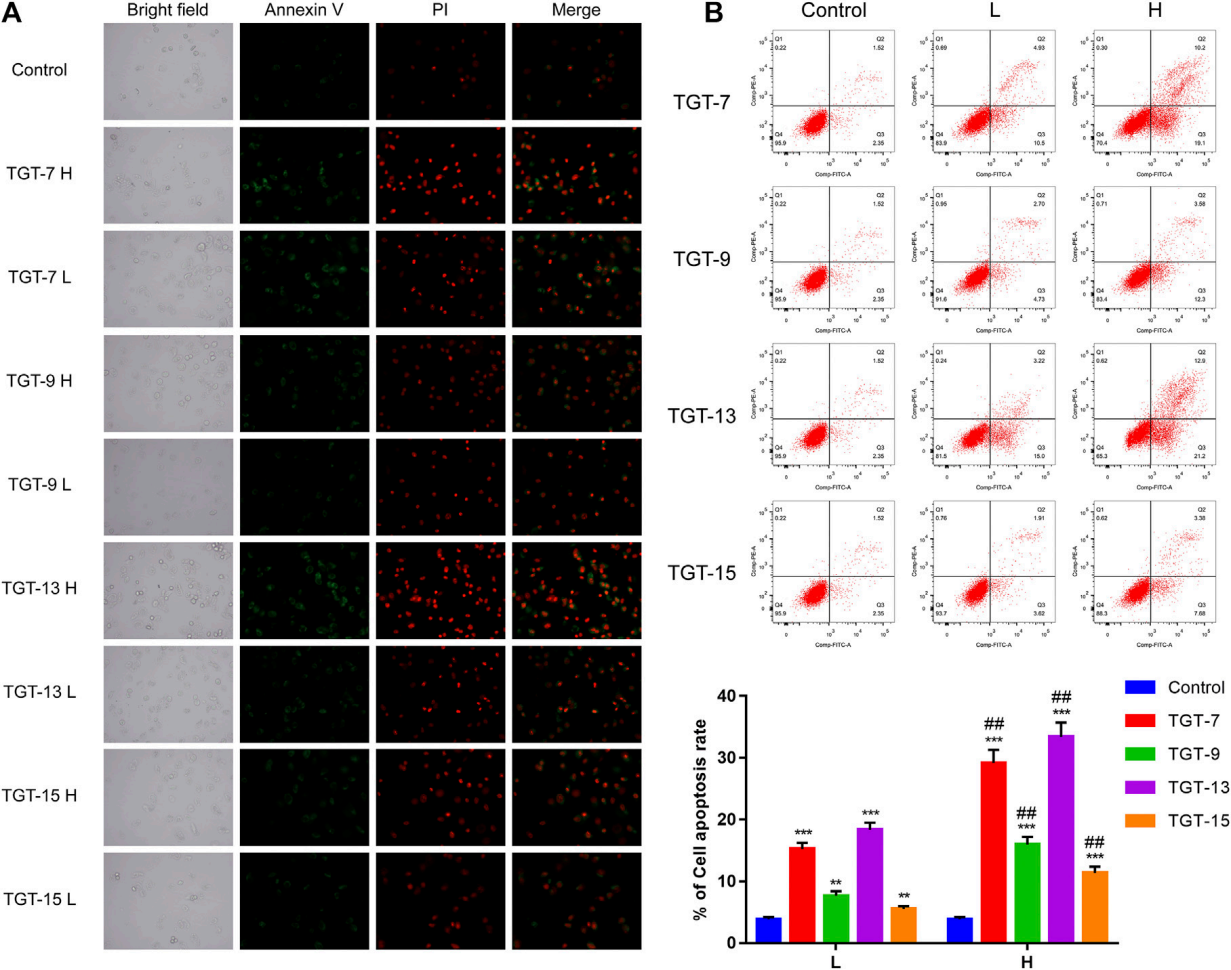
Effects of TGT-7 (28 μm, 56 μm), TGT-9 (44 μm, 88 μm), TGT-13 (29 μM, 58 μm), and TGT-15 (47 μm, 94 μm) on the apoptosis of A549 cells **(A)** Fluoroscope micrographs of apoptotic cells after Annexin V-FITC/PI double staining. Apoptotic cells are indicated in green, whereas necrotic cells are indicated in red (original magnification: ×100) **(B)** Cell apoptosis profiles were assessed using Annexin V-FITC/PI staining and flow cytometry. The biparametric histogram reveals cells in the early (bottom right quadrant) and late apoptotic states (upper right quadrant); viable cells are shown as double-negatives (bottom left quadrant). The numbers show the percentages of each fraction. These results were obtained from three independent experiments, and all data are expressed as the mean ± SD, **p < 0.01, ***p < 0.001 vs. the control group, ^*##*^ *p < 0.01, vs*. the low-dose group (*n* = 3).

Annexin V-FITC/PI double-staining was employed to detect apoptotic cells. The A549 cells were treated with TGT-**7** (28 µm, 56 µm), TGT-**9** (44 µm, 88 µm), TGT-**13** (29 µm, 58 µm), and TGT-**15** (47 µm, 94 µm) for 24 h, and the results revealed higher green fluorescence staining relative to the control group (Figure 11A). In addition, the number of apoptotic cells was determined using flow cytometry, and compared with the control group, the apoptotic cells (the sum of early and late apoptotic cells) in the drug-treated group significantly increased, and an increase in concentration was correlated with a higher number of apoptotic cells (Figure 11B). These data suggested that TGT-**7**, TGT-**9**, TGT-**13**, and TGT-**15** induced apoptosis in A549 cells in a concentration-dependent fashion.

### TGT-7, TGT-9, TGT-13, and TGT-15 Reduce the Mitochondrial Membrane Potential of A549 Cells

**FIGURE 12:**
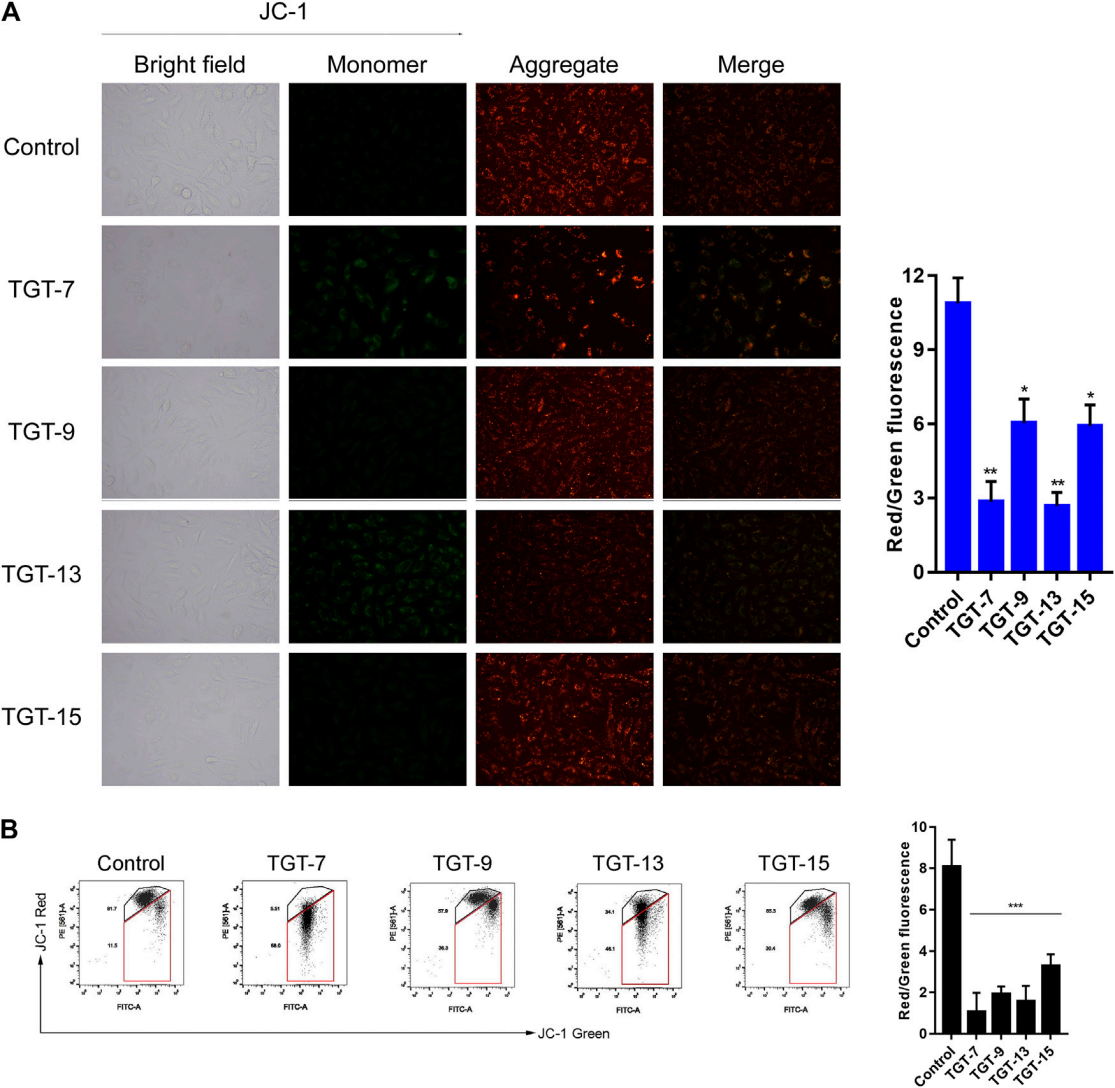
TGT-7, TGT-9, TGT-13, and TGT-15 cause mitochondrial membrane potential dysfunction of A549 cells **(A)** Mitochondrial membrane potential observed using fluorescence miscopy (original magnification ×200) **(B)** Mitochondrial membrane potential observed using flow cytometry. These results were obtained from three independent experiments, and all of the data are expressed as the mean ± SD, *p < 0.05, **p < 0.01, ***p < 0.001 vs. the control group (*n* = 3).

Next, we assessed the effects of TGT-**7**, TGT-**9**, TGT-**13**, and TGT-**15** on A549 cells mitochondrial membrane potential by evaluating changes in the red–green fluorescence ratio after JC-1 staining. The results showed that in A549 cells treated with TGT-**7**, TGT-**9**, TGT-**13**, and TGT-**15**, green fluorescence increased, red fluorescence decreased, and the ratio of red–green fluorescence decreased from 14.24 ± 1.14 to 3.10 ± 0.43, 2.74 ± 0.55, 9.54 ± 0.58, and 9.64 ± 1.10, respectively. Compared to the control group, we observed statistically significant differences (Figure 12A). Then, we verified the findings through flow cytometry experiments, and the results were consistent with the above results (Figure 12B). These results demonstrated that TGT-**7**, TGT-**9**, TGT-**13**, and TGT-**15** treatment resulted in depolarization of the mitochondrial membrane potential.

### TGT-7, TGT-9, TGT-13, and TGT-15 Increase Intracellular ROS Levels in A549 Cells

**FIGURE 13:**
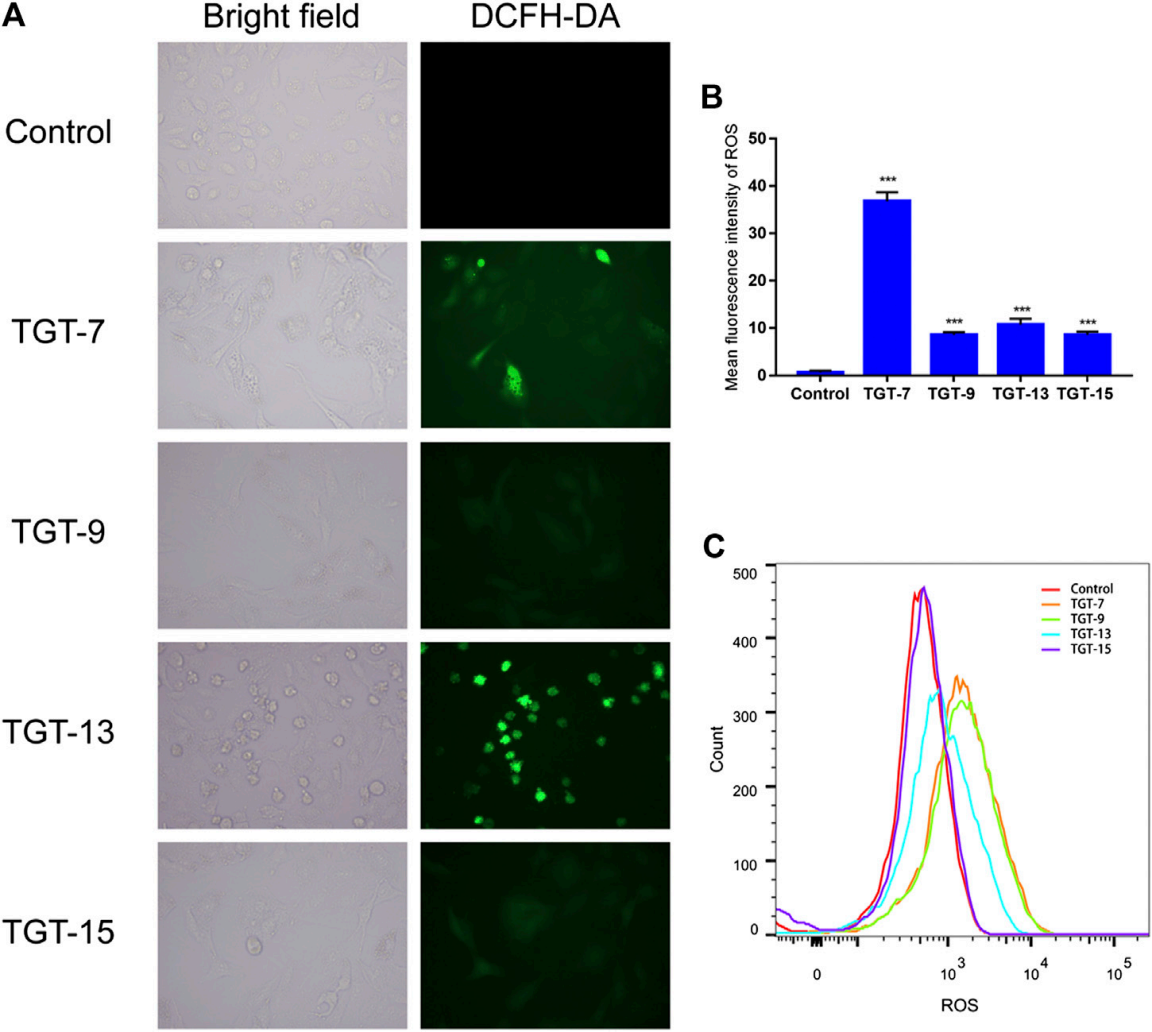
TGT-7, TGT-9, TGT-13, and TGT-15 increase intracellular ROS levels in A549 cells **(A)** and **(B)** Intracellular ROS levels of A549 cells observed using fluorescence microscopy (original magnification ×200) **(C)** Intracellular ROS levels of A549 cells determined by flow cytometry. Results were obtained from three separate experiments, and data are presented as the mean ± SD, ***p < 0.001 vs. the control group (n = 3).

We also examined the effects of TGT-**7**, TGT-**9**, TGT-**13**, and TGT-**15** on ROS production in A549 cells by measuring changes in ROS in A549 cells after DCFH-DA staining. The experimental results showed that after treatment with TGT-**7**, TGT-**9**, TGT-**13**, and TGT-**15**, compared to the control group, green fluorescence in the A549 cells significantly increased. This indicated that these four compounds all caused an increase in intracellular ROS levels (Figures 13A–C). These results demonstrated that TGT-**7**, TGT-**9**, TGT-**13**, and TGT-**15** all induced ROS production in A549 cells, which led to an increase in the concentration of ROS.

### TGT-7, TGT-9, TGT-13, and TGT-15 Modulate Migration and Apoptosis-Related Key Proteins

**FIGURE 14:**
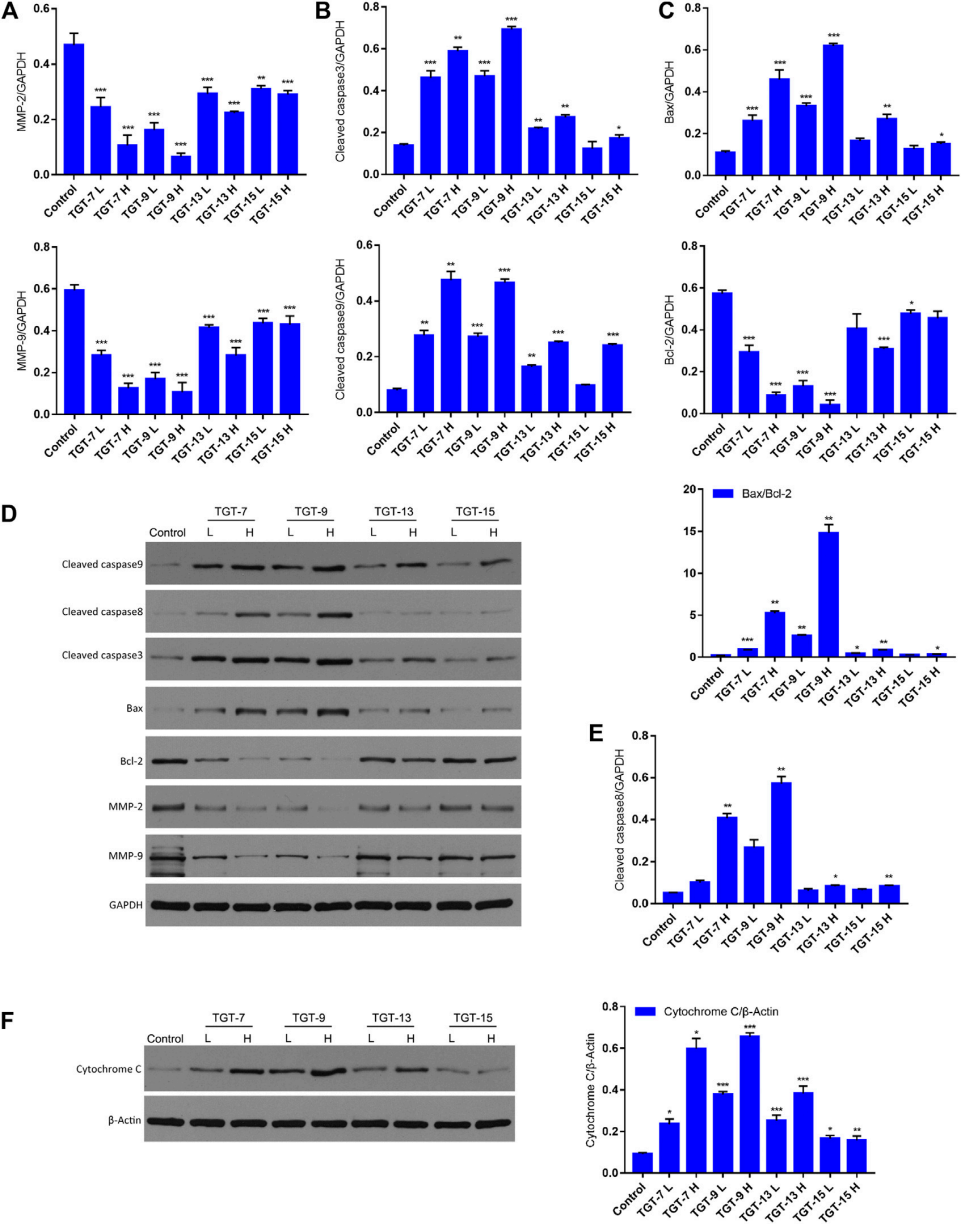
TGT-7, TGT-9, TGT-13, and TGT-15 modulate migration and expression of apoptosis-related key proteins **(A)** The histograms show MMP2 and MMP9 expression **(B)** Histograms showing cleaved caspase-3 and cleaved caspase-9 expression **(C)** Histograms showing Bax and Bcl-2 expression **(D)** Migration and apoptosis-related key proteins were analyzed by Western blotting using *GAPDH* for data normalization **(E)** Histograms showing cleaved caspase-8 expression **(F)** Expression of cytochrome C analyzed by Western blotting and using *β-Actin* for data normalization. These results were obtained from three independent experiments, and all of the data are expressed as the mean ± SD, *p < 0.05, **p < 0.01, ***p < 0.001 vs. the control group (*n* = 3).

To further clarify the potential molecular mechanisms of TGT-**7**, TGT-**9**, TGT-**13**, and TGT-**15** inhibiting the growth of A549 cells, we next examined key proteins that were closely related to migration and apoptosis. The results showed that TGT-**7**, TGT-**9**, TGT-**13**, and TGT-**15** decreased MMP-2 and MMP-9 expression in A549 cells. However, this gradually decreased with increasing concentrations and showed a dose-dependent manner. In addition, we examined the expression patterns of cytochrome C, Bax, Bcl-2, cleaved caspase-3, and cleaved caspase-9 (Figures 14A–C). The results showed that the high and low concentrations of TG-T-**7**, TGT-**9**, TGT-**13**, and TGT-**15** increased the expression of cytochrome C, and the high and low concentrations of TGT-**7**, TGT-**9**, and TGT-**13** and the high concentrations of TGT-**15** increased the expression of caspase-9 and caspase-3, indicating that all four compounds could promote the release of mitochondrial cytochrome C and TGT-7, TGT-9, and TGT-13 could activate caspase-9 and then activate caspase-3 to induce apoptosis. TGT-**7**, TGT-**9**, TGT-**13**, and TGT-**15** could increase the expression of cleaved caspase-8 at high concentrations, which suggested that TGT-**7**, TGT-**9**, TGT-**13**, and TGT-**15** could induce apoptosis in the mitochondrial pathway and possibly through the death receptor pathway.

## Discussion

*Marsdeniae tenacissimae* Caulis extract (Xiao-Ai-Ping) has been clinically used in the treatment of malignant tumors, such as liver cancer, leukemia, lung cancer, etc. Some scholars have specifically used the MTT method to detect the effect of extracts of TGT on the proliferation of normal human lymphocyte cells induced by concanavalin A (ConA) and lipopolysaccharide (LPS). Results have shown that TGT extracts have no obvious cytotoxic effects on normal immune cells and hematopoietic stem cells *in vitro*, but can promote the proliferation of T and B cells, which were closely related to tumor patients’ immunity capacity against the aforementioned diseases (Chen et al., 2010; Wang et al., 2017; Zheng et al., 2017; Zhan et al., 2019). Studies have found that C21 steroidal glycosides in *Marsdeniae tenacissimae* Caulis were the main active components. Intriguingly, extensive research demonstrated that C21 steroidal glycosides harbor anti-tussive, anti-asthma, and antitumor activities (Pang et al., 2018; Wang et al., 2018).

Our preliminary research showed the ethyl acetate extracted from a TGT extract had the strongest growth inhibition against A549 cells *in vitro*. We also demonstrated the anti-tumor activity of this extract *in vivo*. Results from others showed that LLC tumor-bearing male C57BL/6 mice in a cisplatin group lost more weight than others, indicating *M. tenacissima* extracts did not cause severe side effects while reducing tumor size (Xu, 2018; Hu et al., 2020). Therefore, this study was aimed at separating the components of an ethyl acetate fraction in order to identify compounds with strong anti-NSCLC activity.

First, we isolated and purified the ethyl acetate part of *Marsdeniae tenacissimae* Caulis. A total of 19 compounds were isolated from an ethyl acetate fraction, 15 of which were C21 steroidal glycosides (Figure 1). At the same time, we elucidated their structures, which included 2 novel compounds, namely, 11*α*-*O*-benzoyl-12*β*-*O*-tigloyltenacigenin B **15**) (Figure 2, Table 1) and sodium 5-hydroxy-4-( ( (2*S*,3*R*,4*S*,5*R*,6*R*)-5-hydroxy-3-(((2*R*,3*R*,4*R*,5*R*,6*S*)-5-hydroxy-6-((2-hydroxy-4,6-dimethoxy-6-oxohexan-3-yl)oxy)-4-methoxy-2-methyltetrahydro-2H-pyran-3-yl)oxy)-4-methoxy-6-methyltetrahydro-2H-pyran-2-yl)oxy)-3-methoxyhexanoate **18**) (Figure 3, Table 1), and 17 known compounds (**1**–**14, 17, 19, 20**) (Figure 4, Supplementary Tables S1,S2). Among all the isolated monomeric compounds, most were C21 steroids. These findings indicated that C21 steroidal saponins might be the effective anti-tumor components of *Marsdeniae tenacissimae* Caulis*.*

Second, we evaluated all of the isolated compounds against A549 cells using an *in vitro* assay (Table 2). The results indicated that the six types of steroidal saponins had inhibitory effects against A549 cells *in vitro,* as their IC50 values were less than 100 µm. At the same time, we observed that A549 cells were sensitive to these four C21 steroidal glycosides in a dose-dependent manner after 24 h of drug stimulation (Xu, 2018). Moreover, we assessed the cytotoxic effect of the four C21 steroidal glycosides against BEAS-2B normal human pulmonary epithelial cells using trypan blue dye exclusion assay, and the results showed that they had no obvious cytotoxicity against BEAS-2B cells.

Next, we analyzed the structure–activity relationships of these isolated compounds. Their structures were very similar, but their activities varied widely in A549 cells *in vitro* (Table 2). The main differences in their structure were due to 11-position and 12-position substituents. When the 12-position was acetyl-substituted, these compounds were essentially inactive in A549 cells *in vitro,* such as compounds **1, 2, 3, 4,** and **19**. When the 12-position was substituted by benzoyl, they had the best activity, such as compounds **7**, **9**, and **13**, followed by methylcrotonyl substitution (compounds **8**, **10**, and **15)**. Therefore, we postulated that the steroidal compounds that acted on the activity of A549 cells *in vitro* were mainly C-12 substituents, and when benzoyl was substituted, the inhibitory effect against A549 cells was the strongest, whereas the effect of the sugar chain was minimal. Differences in activity were attributable to variations in substituents at the C-12 position. This finding might be further utilized as a reference for structural modifications to identify active compounds.

Traditional Chinese medicine (TCM) network pharmacology is a novel research approach that predicts target profiles as well as the pharmacological actions of various herbal compounds and identifies drug–gene–disease comodule correlations to determine the integrated rules and network regulatory effects of different herbal formulae (Wu et al., 2016). This provides a new paradigm for elucidating the pharmacodynamic substance basis and unraveling the mechanisms of action of TCM (Lee et al., 2019; Zhang et al., 2019).

In order to better determine the best anti-NSCLC substances and potential mechanisms of *M. tenacissimae*, we preliminarily used network pharmacology to screen six active compounds. Network pharmacological analysis showed that steroidal saponins imparted anti-cancer effects mainly *via* 18 targets that were closely related to the PI3K/AKT, RAS/RAF/MEK/ERK, VEGF, and MAPK signaling pathways. These pathways were associated with angiogenesis, cell cycle change, migration, invasion, and cancer cell apoptosis (Figures 6B, 7A). According to the size of the node and degree in the network of the NSCLC-compound-target and PPI (Figure 7B), we determined the anti-NSCLC effect of a compound and the importance of a protein in the development of NSCLC so as to provide references for follow-up research focused on deepening our understanding of this mechanism.

One of the major goals of cancer treatment is to disrupt tumor cell proliferation *via* cell cycle progression blockage (Evan and Vousden, 2001; Wang et al., 2020). Currently, a number of chemotherapeutic drugs can block tumor cells in the G0/G1, S, or G2/M phases and thus can achieve the aim of inhibiting tumor cell proliferation (Schwartz and Shah, 2005; Chen et al., 2016). Based on the results of literature research and our above network pharmacological analysis, our experiment demonstrated that the four C21 steroidal glycosides (TGT-**7**, TGT-**9**, TGT-**13**, and TGT-**15**) also blocked A549 cells at the G0/G1 phase and prevented cells from progressing toward the S (DNA replication) and M (cell division) phases, as well as decreased the rates of cell growth and proliferation (Figure 10). Cell proliferation was closely linked to the cell cycle, which normally operates in an orderly manner under the supervision of cell cycle-related genes, and thus cancer occurs when cell cycle errors cause cells to proliferate (Yang et al., 2008).

MMP-2 and MMP-9 have been strongly linked to angiogenesis, invasion, and metastasis in tumor cells (Webb et al., 2017). Our study illustrated that four C21 steroidal glycosides could decrease the expression of MMP-2 and MMP-9 in A549 cells. Moreover, with increasing concentration, their expression slowly decreased in a dose-dependent manner (Figures 14A,D).

Apoptosis induction is considered an essential mechanism of antitumor therapeutics (Stepczynska et al., 2001). Previous studies have revealed that anticancer agents impart anti-proliferative effects using two distinct apoptosis pathways that involve mitochondria or death receptors (Kuo et al., 2018; Han et al., 2019). Specifically, the mitochondria-associated pathway is a classic intrinsic pathway that is caused by ROS overproduction, which in turn results in the depletion of △φin (Shi et al., 2014; Wang et al., 2019). Generally, various protein molecules participate in regulating the mitochondrial apoptotic pathway such as pro-apoptotic members (Bax and Bad) and anti-apoptotic members (Bcl-2 and Bcl-xl) (Dong et al., 2019). Moreover, the pathway activated specific pivotal proteinases such as initiator caspase-9 and effector caspase-3 and subsequently resulted in DNA fragmentation as well as nuclear PARP degradation during apoptosis (Duangprompo et al., 2016; Chao et al., 2018). Therefore, Annexin V-FITC/PI staining was conducted to detect apoptotic alterations that may be related to A549 cells cytotoxicity. We detected changes in fluorescence intensities after A549 cells were stained by Annexin V-FITC/PI and observed that the four C21 steroidal glycosides influenced A549 cells apoptosis. In this experiment, we discovered that the rate of cell apoptosis increased in the drug treatment group as measured by flow cytometry and was positively correlated with the drug concentration. Therefore, the results indicated that the four C21 steroidal glycosides could effectively promote A549 cells apoptosis (Figure 11).

Mitochondria serve as the regulatory center of endogenous pathways of apoptosis. Mitochondrial status can be interrogated by measuring the mitochondrial membrane potential (Green and Reed, 1998). Based on this point, our experimental results showed that the four C21 steroidal glycosides could reduce the mitochondrial membrane potential, indicating that this might induce A549 apoptosis *via* endogenous pathways of the mitochondria (Figure 12). ROS are mainly produced in the mitochondria, and ROS overproduction could lead to lipid overoxidation of the mitochondrial membrane, further influencing the mitochondrial membrane potential and triggering the release of cytochrome C, which, in turn, induces endogenous apoptosis (Zhao and Xu, 2001). Some studies have shown that ROS can also cause exogenous apoptosis mainly by increasing the sensitivity of tumor cells to Fasl, then activating caspase-8 to mediate exogenous apoptosis through the death receptor Fas/Fasl pathway (Shang et al., 2016). In our experiment, the effect of the four C21 steroidal glycosides on the ROS level was assessed in A549 cells. The experimental results indicated that the green fluorescence of every drug group was significantly more intense relative to the control group after TGT-**7**, TGT-**9**, TGT-**13**, and TGT-**15** treatment. Thus, this experiment clearly showed that TGT-**7**, **9**, **13**, and **15** could raise the level of ROS in A549 cells and then induce cell apoptosis (Figure 13).

The cytochrome C-mediated mitochondrial apoptosis pathway is controlled by the Bcl-2 protein family, and in terms of apoptosis, the Bcl-2 family consists of two members, anti-apoptotic protein (Bcl-2) and pro-apoptotic protein (Bax). The ratio of these two is usually an indicator of apoptosis (Adams and Cory, 1998; Li et al., 2017). In our studies, the expression level of Bax increased with increasing concentrations of TGT-**7**, TGT-**9**, TGT-**13**, and TGT-**15**, whereas the Bcl-2 expression level showed the opposite pattern, i.e., it was negatively correlated with dose: an increase in the Bax/Bcl-2 ratio induced the release of cytochrome C that in turn induced cell apoptosis (Figures 14B–D,F). Caspase-8 is a major apoptosis factor in the death receptor pathway (Munoz-Pinedo and Lopez-Rivas, 2018). The four C21 steroidal glycosides at high concentrations could increase cleaved caspase-8 protein expression, suggesting that the four C21 steroidal glycosides could induce apoptosis *via* the death receptor pathway in addition to the mitochondrial pathway (Figures 14D,E).

## Conclusion

**FIGURE 15:**
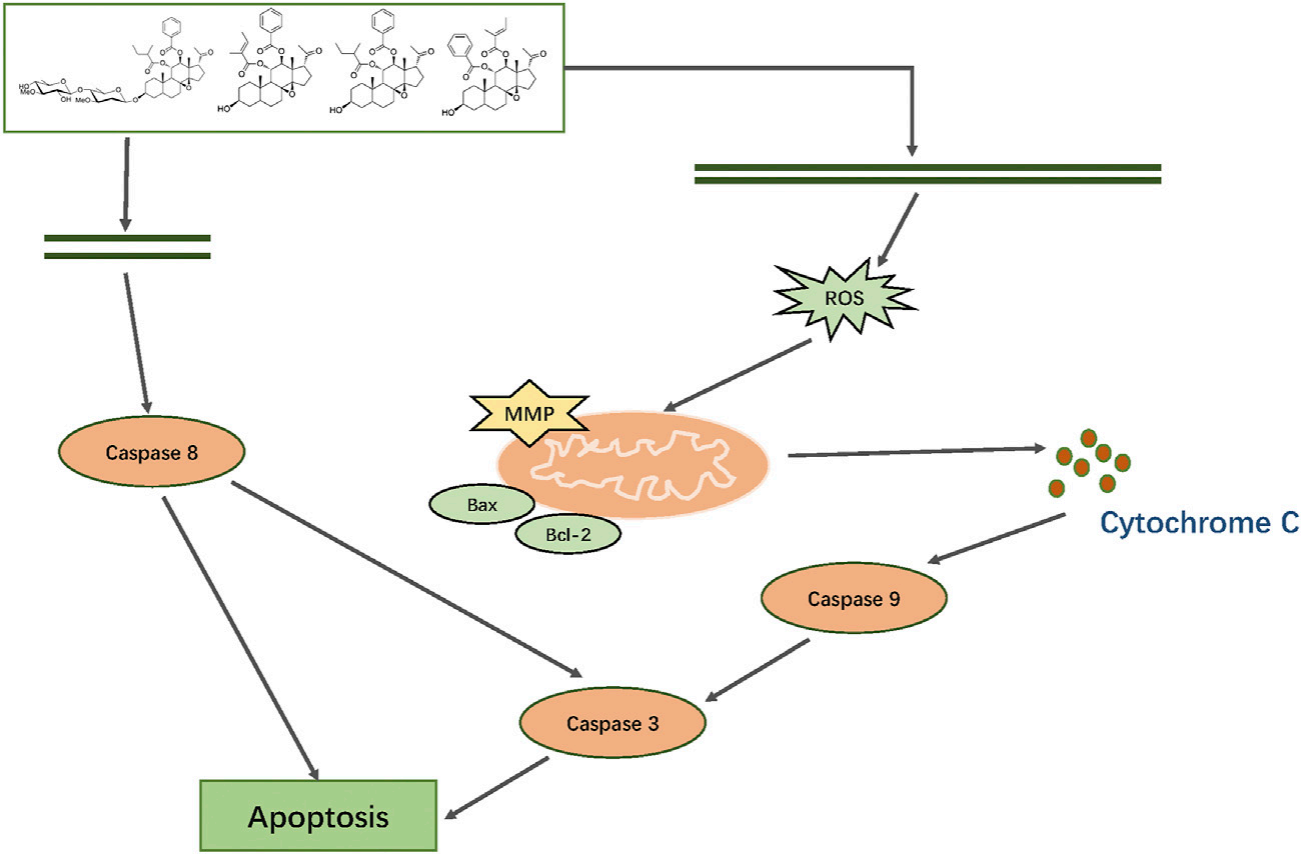
Mechanism by which TGT-7, TGT-9, TGT-13, and TGT-15 isolated from *Marsdeniae tenacissimae* disrupt A549 cells growth and induce apoptosis *via* mitochondrial and death receptor pathways.

In summary, we first isolated and characterized 19 major constituents of *Marsdeniae tenacissimae* Caulis by ^1^H-NMR, ^13^C-NMR and DEPT, 2D NMR (^1^H-^1^HCOSY NOESY, HSQC and HMBC) spectra. Then, we demonstrated that the six main active components of ethyl acetate dramatically suppressed A549 cancer cell proliferation. Furthermore, network pharmacology analysis of the six compounds of *Marsdeniae tenacissimae* Caulis revealed that possible targets were mainly related to the positive regulation of ROS-associated metabolic processes, as well as intrinsic apoptotic pathways. Next, a series of cellular tests verified the results of the network pharmacology prediction. The four C21 steroidal glycosides (TGT-**7**, TGT-**9**, TGT-**13**, and TGT-**15**) disrupted A549 cells migration and invasion *via* downregulation of MMP-2 and MMP-9 expression. We also found that C21 steroidal glycosides of *Marsdeniae tenacissimae* Caulis triggered apoptosis of A549 cells through a mitochondrial-mediated pathway *via* upregulation of Bax and downregulation of Bcl-2 expression, thus releasing cytochrome C and finally activating caspase-3 and caspase-9. At the same time, the four C21 steroidal glycosides also activated caspase-8, which activated the death receptor pathway to promote apoptosis. The four C21 steroidal glycosides disrupted A549 growth and triggered apoptosis *via* mitochondrial and death receptor pathways (Figure 15).

At present, there are many studies on the treatment of cancer by *Marsdeniae tenacissimae* Caulis and its clinical preparation Xiao-Ai-Ping injection, but these studies have not excavated its pharmacodynamic material basis. In our research, we have defined the antitumor compounds as well as their mechanisms, which can be potentially employed as a therapeutic option for the treatment alone of NSCLC or in combination with anticancer chemical drugs to reduce their toxicity and side effects. In addition, we also analyzed the structure-activity relationship of these isolated compounds, which provides experimental basis for the development of clinical anticancer drugs or to improve the clinical efficacy of existing anticancer chemical drugs by structural modification in the future. Due to the approaching completion time, the relevant targets and pathways predicted by the network pharmacology in this study on the anti-NSCLC caused by *M. tenacissima* have not been fully verified, which will be studied in our later research.

## Data Availability

All datasets generated for this study are included in the article/Supplementary Material.

## Footnotes

1
https://www.ncbi.nlm.nih.gov/pubmed

2
http://www.chemspider.com/

3
http://www.hmdb.ca/

4
https://metlin.scripps.edu/landing_page.php?pgcontent=mainPage

5
https://sso.cas.org/as/kNab3/resume/as/authorization.ping

6
http://www.swisstargetprediction.ch/

7
http://stitch.embl.de/

8
http://www.disgenet.org/

9
https://omim.org/

10
https://www.genome.jp/kegg/ligand.html

11
https://david.ncifcrf.gov/relatedGenes.jsp
