# Supplementary material for: A Combined Phytochemistry and Network Pharmacology Approach to Reveal Potential Anti-NSCLC Effective Substances and Mechanisms in Marsdenia tenacissima (Roxb.) Moon (Stem)
Source: Front Pharmacol. 2021 Apr 29;12:518406. doi: 10.3389/fphar.2021.518406 (PMC8117745; doi:10.3389/fphar.2021.518406)
Supplement: Supplementary file 1 [file datasheet1.zip › Data Sheet/Supplementary Material/Figure S2.pdf]

Figure S2. ^{1}H-NMR, ^{13}C-NMR, 1H-1H COSY ,HMBC, HSQC and NOESY Spectrum of Compound 18

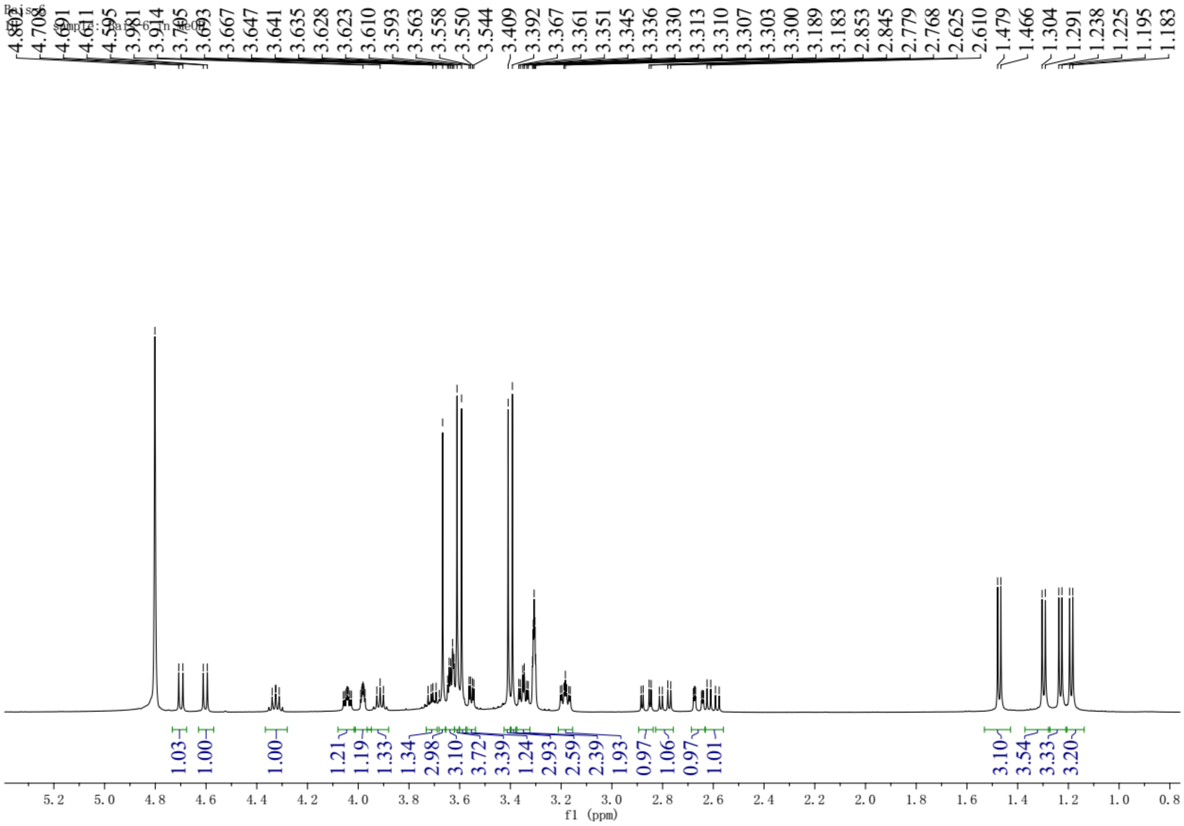

^{1}H-NMR Spectrum of Compound 18

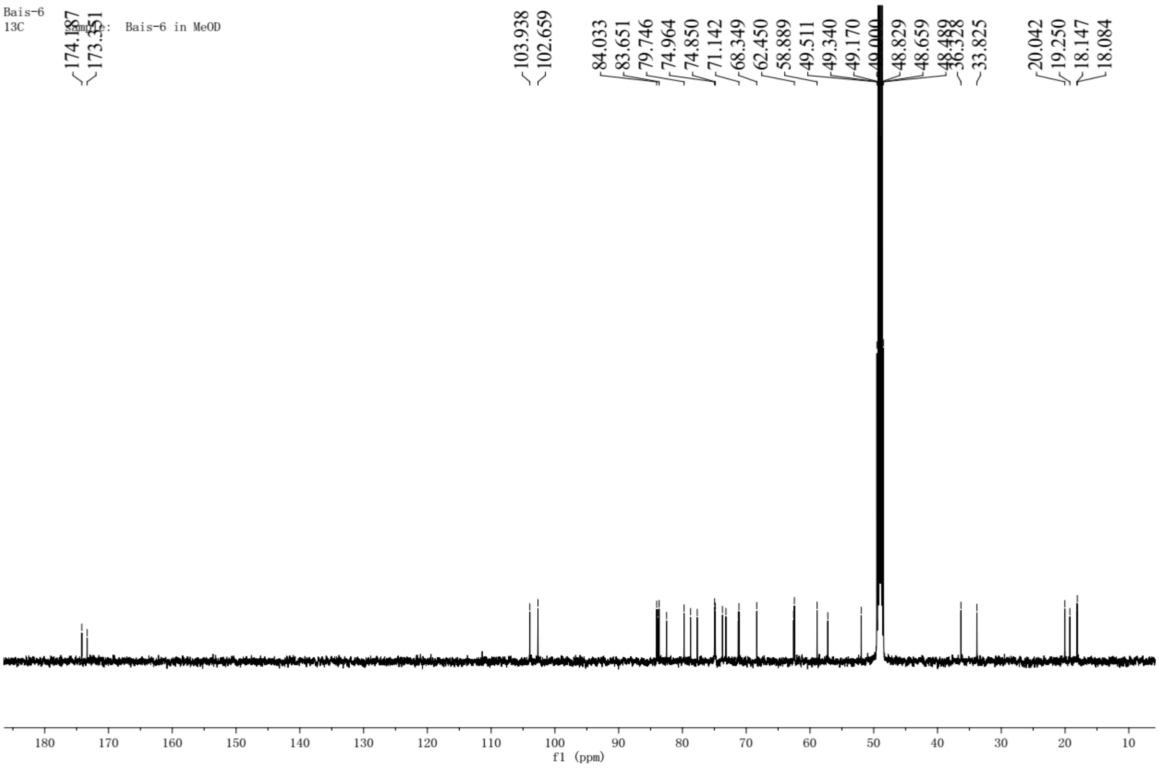

^{13}C-NMR Spectrum of Compound 18

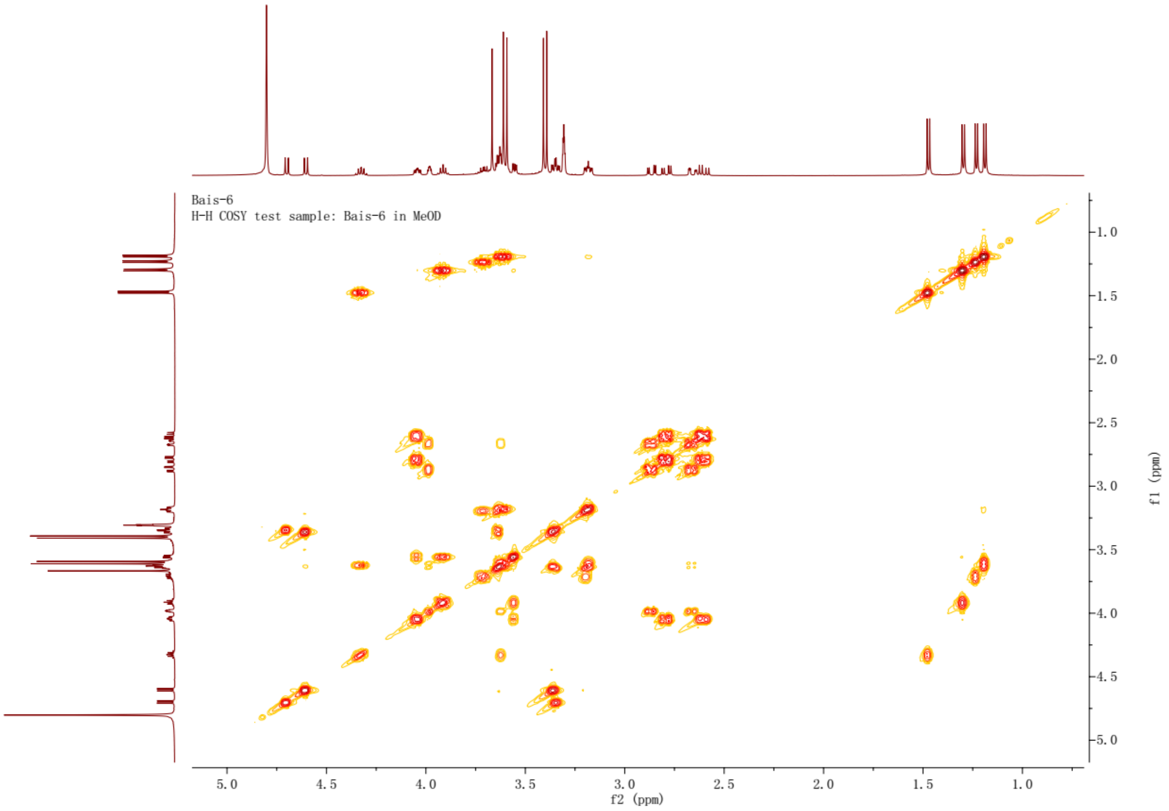

^{1}H-^{1}H COSY Spectrum of Compound 18

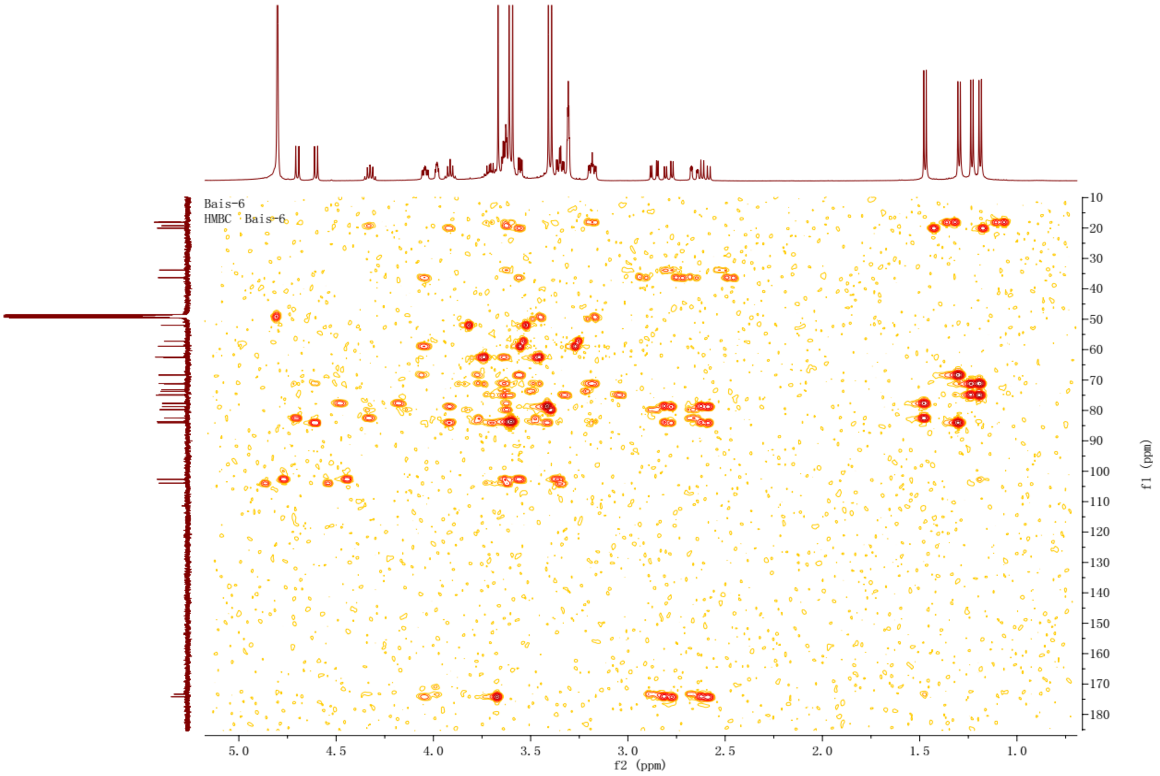

HMBC Spectrum of Compound 18

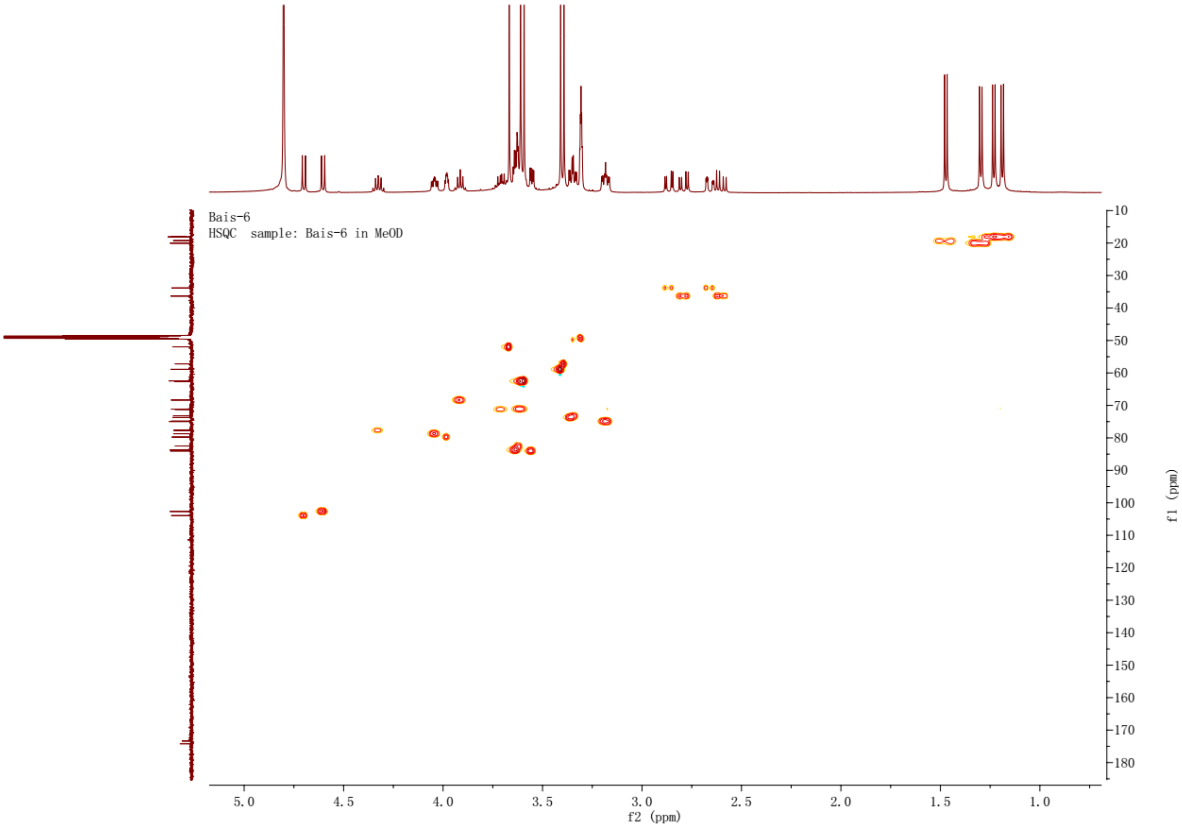

HSQC Spectrum of Compound 18

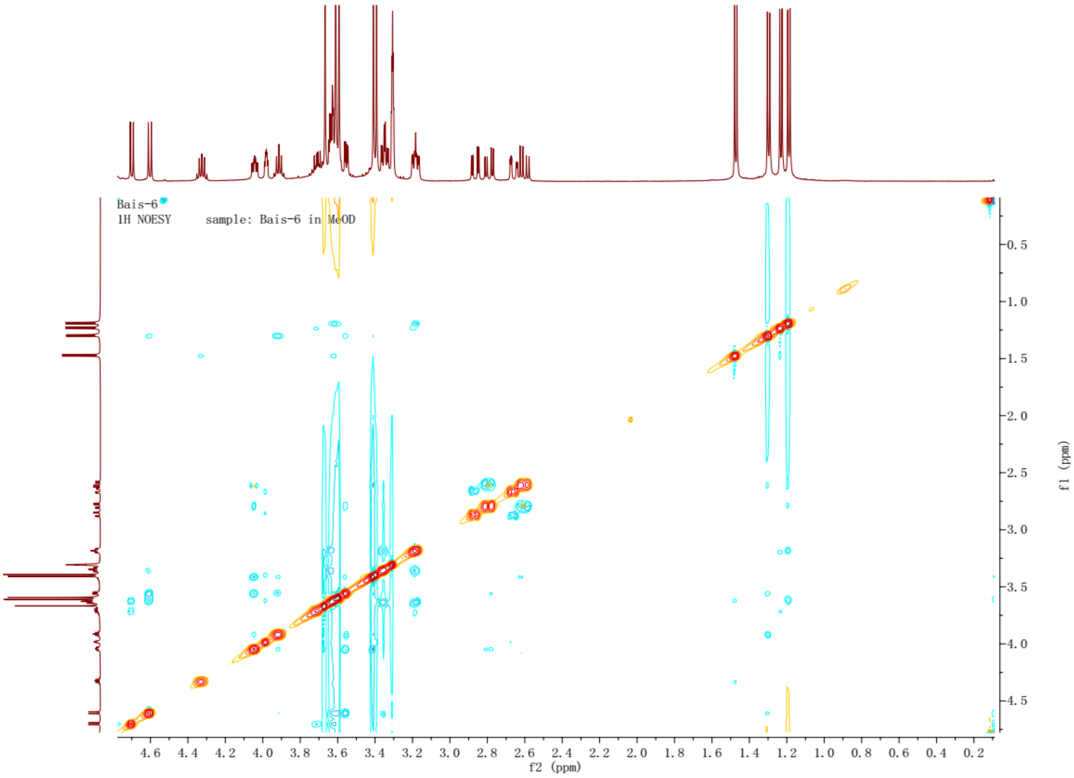

NOESY Spectrum of Compound 18
