# Supplementary material for: A Combined Phytochemistry and Network Pharmacology Approach to Reveal Potential Anti-NSCLC Effective Substances and Mechanisms in Marsdenia tenacissima (Roxb.) Moon (Stem)
Source: Front Pharmacol. 2021 Apr 29;12:518406. doi: 10.3389/fphar.2021.518406 (PMC8117745; doi:10.3389/fphar.2021.518406)
Supplement: Supplementary file 1 [file datasheet1.zip › Data Sheet/Supplementary Material/File S1.pdf]

File S1

Seventeen known compounds (1-14, 17, 19, 20) were isolated from the ethyl acetate extract of *Caulis Marsdeniae Tenacissima* and their structural data attribution.

Compound 1：Tenacissoside I

White powder (methanol), ^{1}H-NMR (MeOD): δ_H1.08 (3H, s, 19-CH_3), 1.10 (3H, s, 18-CH_3), 1.19 (3H, d, *J*=5.8 Hz, Ole-6-CH_3),1.58 (3H, s, 2″-CH_3), 2.16 (3H, s, 21-CH_3), 3.03 (1H, br d, *J*=7.2 Hz, H-17β), 3.40 (3H, s, Ole-3-OCH_3), 3.60 (3H, s, Allo-3-OCH_3), 4.59 (1H, dd, *J*=8.4, 1.6 Hz, Ole-H-1), 4.70 (1H, d, *J*=8.4 Hz, Allo-H-1), 5.01 (1H, d, *J*=10.2Hz, H-12α), 5.35 (1H, t, *J*=10.2 Hz, H-11β), 7.49 (2H, t, *J*=7.8 Hz, H-4′, 6′), 7.60 (1H, t, *J*=7.2 Hz, H-5′), 7.94 (2H, d, *J*=7.2 Hz, H-3′,7′). ^{13}C-NMR: Table S1, S3.

Compound 2：Tenacissoside G

White powder (methanol), ^{1}H-NMR (MeOD): δ_H1.03 (3H, s, 19-CH_3), 1.08 (3H, s, 18-CH_3), 1.23 (3H, d, *J*=6.6 Hz, Ole-6-CH_3), 1.35 (3H, d, *J*= 6.0 Hz, Allo-6-CH_3), 1.78 (3H, d, *J*=7.2 Hz, 4′-CH_3), 1.79 (3H, s, 2″-CH_3)，1.83 (3H, s, 5′-CH_3), 2.16 (3H, s, 21-CH_3), 3.03 (1H, d, *J*=7.2 Hz, H-17β), 3.40 (3H, s, Ole-3-OCH_3), 3.60 (3H, s, Allo-3-OCH_3), 4.59 (1H, dd, *J*=8.4, 1.6 Hz, Ole-H-1), 4.71 (1H, d, *J*=7.8 Hz, Allo-H-1) , 5.01 (1H, d, *J*=10.2 Hz, H-12α), 5.35 (1H, t, *J*=10.2 Hz, H-11β), 6.78 (1H, m, H-3′). ^{13}C-NMR: Table S1, S3.

Compound 3：11α-O- Benzoyl -12β-O-acetyltenacigenin B

White powder (methanol), ^{1}H-NMR (MeOD)：δ_H1.09 (3H, s, 19-CH_3) , 1.14 (3H, s, 18-CH_3), 1.61 (3H, s, 2″-CH_3), 2.16 (3H, s, 21-CH_3), 3.04 (1H, d, *J*=7.8 Hz, H-17β) , 5. 14 (1H, d, *J* =10.2 Hz, H-12α), 5.57 (1H, t, *J*=10.2 Hz, H-11β), 7.48 (2H, t, *J*=7.8 Hz, H-4′,6′) , 7.62 (1H, t, *J*=7.2 Hz, H-5′) , 7.94 (2H, d, *J*=7.8 Hz, H-3′,7′). ^{13}C-NMR: Table S1.

Compound 4：11α-O-Tigloyl-12β-O-acetyltenacigenin B

Colorless needle crystals (methanol), ^{1}H-NMR (MeOD)：δ_H 1.03 (3H, s, 19-CH_3) , 1.07 (3H, s, 18-CH_3), 1.77 (3H, s, 2″-CH_3), 1.79 (3H, d, *J*=6.6 Hz, 4′-CH_3) 1.83 (3H, s, 5′-CH_3) , 2.16 (3H, s, 21-CH_3), 2.98 (1H, d, *J*=7.8 Hz, H-17β), 5.01 (1H, d, *J*=10.2 Hz, H-12α), 5.36 (1H, t, *J*=10.2 Hz, H-11β), 6.81 (1H, q, *J*=7.2 Hz, H-3′). ^{13}C-NMR:

Table S1.

Compound 5： β -sitosterol

White needle crystals ($CDCl_3$)，^{1}H-NMR ($CDCl_3$): δ_H 0.68 (3H, s, H-18), 0.77-0.86 (9H, m, H-26, 28, 29), 1.02 (3H, s, H-19) , 0.96(3H, m, H-21) , 0.97 (2H, m, H-22) , 5. 38(1H, m, H -10) , 3. 51(1H, m, H-3) , 1.01-2.24(28H, m, H-1, 2, 4, 7, 8, 9, 11, 12, 14, 15, 16, 17, 20, 23, 24, 25, 27; ^{13}C-NMR ($CDCl_3$): δc37. 4(C-1), 31.9 (C-2), 72.0 (C-3), 42.5(C-4), 140.9(C-5), 121.9(C-6), 21.8(C-7), 32.0(C-8), 50.3(C-9), 37.2(C-10), 21.4(C-11), 40.0(C-12), 42.4(C-13), 57.5(C-14), 24.5(C-15), 31.8(C-16), 57.0(C-17) , 12.1(C-18), 19.5(C-19), 36.7(C-20) , 18.9(21), 34.5(C-22), 26.5(C-23) , 46.0(C-24) , 23.7(C-25), 12.0(C-26), 28.4(C-27), 20.0(C-28) , 19.2(C-29).

Compound 6： Daucosterol

White powder (chloroform-methanol)，^{1}H-NMR (DMSO): δ_H0.64(3H, s, CH_3), 0. 92(3H, s, CH_3), 0. 89(3H, d, *J*= 7.8 Hz, CH_3), 0.94(3H, m, CH_3), 0.98(3H, d, *J*=7.8Hz, CH_3), 0.82(3H, t, *J* =8.4 Hz, CH_3), 3.40(1H, m, H-3), 5.32(1H, m, H-6); ^{13}C-NMR (DMSO): δc38.7(C-1), 31.1(C-2), 78.8(C-3), 40.5(C-4), 142.3(C-5), 123(C-6), 33.3(C-7), 33.2(C-8), 51.5(C-9), 38.1(C-10), 22.6(C-11), 40.3(C-12), 43.7(C-13), 58.0(C-14), 25.7(C-15), 29.9(C-16), 57.3(C-17), 13.6(C-18), 20.8(C-19), 38.1(C-20), 20.5(C-21), 35.4(C-22), 27.5(C-23), 47.0(C-24), 30.6(C-25), 21.0(C-26), 21.6 (C-27), 24.5(C-28), 13.5(C-29). Glu:102.7(1), 75.3(2), 78. 8(3), 72.0(4), 78. 6(5), 63.0(6).

Compound 7：Marsdenoside C

White powder (methanol), ^{1}H-NMR ($CDCl_3$): δ_H0.55 (3H, t, *J*=7.2 Hz, 4′-CH_3), 0.85 (3H,d, *J*=7.2 Hz, 5′-CH_3), 1.09 (3H, s, 19-CH_3), 1.16 (3H, s, 18-CH_3), 1.26 (3H, d, *J*=6.0 Hz, Allo-6-CH_3), 1.37(3H, d, *J*=3.6 Hz, Ole-6-CH_3), 1.48 (1H, m, Ole-Ha-2), 2.08 (1H, d, *J*=10.2 Hz, H-9), 2.28(3H, s, 21-CH_3), 2.33 (1H, ddd, *J*=12.6, 4.8, 1.7 Hz, Ole-He-2), 2.99 (1H, d, *J*=7.2 Hz, H-17β), 3.35 (2H, m, Ole-H-4, 5),3.38(3H, s, Ole-3-OCH_3), 3.41 (1H, m, Ole-H-3), 3.49(1H, dd, *J*=8.2, 2.8 Hz, Allo-H-2), 3.56 (1H, m, AlloH-5), 3.64 (1H, m, H-3), 3.67 (3H, s, Allo-3-OCH_3),3.80 (1H, t, *J*=2.9 Hz, Allo-H-3), 4.59 (1H, d, *J*=9.6Hz,1.8Hz, Ole-H-1), 4.80 (1H, d, *J*=8.4 Hz, Allo-H-1), 5.25 (1H, d, *J*=10.2 Hz, H-12α), 5.54 (1H,t, *J*=10.2 Hz, H-11β), 7.42 (2H, t, *J*=7.2 Hz, H-4″,6″), 7.55 (1H, t, *J*=7.4 Hz, H-5″), 7.96 (2H, d, *J*=7.8 Hz, H-3″,7″). ^{13}C-NMR:

Table S1, S3.

Compound 8：Marsdenoside A

Colorless needle crystals (methanol), ^{1}H-NMR ($CDCl_3$): δ_H0.79(3H, t, J=7.2 Hz, 4′-CH_3), 0.97 (3H, d, J=6.9 Hz, 5′-CH_3), 1.05 (3H, s, 19-CH_3), 1.09 (3H, s, 18-CH_3), 1.26 (3H, d, J=6.0 Hz, Allo-6-CH_3), 1.37 (3H, d, J=4.2 Hz, Ole-6-CH_3), 1.49 (1H, m, Ole-Ha-2), 1.74 (3H, br s, 5″-CH_3), 1.76 (3H, d, J=6.6 Hz, 4″-CH_3), 2.02 (1H, d, J=9.6 Hz, H-9), 2.08 (1H, q, J=6.6 Hz, H-2″), 2.23(3H, s, 21-CH_3), 2.31 (1H, dd, J=11.7, 4.0 Hz, Ole-He-2), 2.92 (1H, d, J=7.2 Hz, H-17β), 3.18 (1H, br d, J=9.5Hz, Allo-H-4), 3.35 (1H, t, J=6.6 Hz, Ole-H-4), 3.36 (1H, m, Ole-H-5), 3.37 (3H,s, Ole-3-OCH_3), 3.41 (1H, m, Ole-H-3), 3.48 (1H, dd, J=7.2, 2.6 Hz, Allo-H-2), 3.56 (1H, m, Allo-H-5), 3.63 (1H, m, H-3), 3.67 (3H, s, Allo-3-OCH_3), 3.79 (1H, s, Allo-H-3), 4.58 (1H, dd, J=9.7, 1.6 Hz,Ole-H-1), 4.80 (1H, d, J = 7.8 Hz, Allo-H-1), 5.04 (1H,d, J=9.6 Hz, H-12α), 5.41 (1H, t, J=9.6 Hz, H-11β), 6.79 (1H, q, J=6.0 Hz, H-3″). ^{13}C-NMR: Table S1, S3.

Compound 9：11-α-O-tigloyl-l2β-O-benzoyltenacigenin B

White powder (methanol)，^{1}H-NMR ($CDCl_3$): δ_H 1.11 (3H, s, 19-CH_3), 1.19 (3H, s, 18-CH_3), 1.43 (3H, s, 5′-CH_3), 1.47 (3H, d, J=7.2Hz, 4′-CH_3), 2.09 (1H, d, J=10.8 Hz, H-9), 2.27 (3H, s, 21-CH_3), 3.00 (1H, d, J=7.2 Hz, H-17β), 3.60 (1H, m, H-3), 5.26 (1H, d, J=10.2 Hz, H-12α), 5.60 (1H, t, J=10.2 Hz, H-11β), 6.79 (1H, q, J=6.0 Hz, H-3′), 7.38 (2H, t, J=7.8 Hz, H-4″,6″), 7.52 (1H, t, J=7.2 Hz, H-5″), 7.90 (2H, d, J=7.8Hz, H-3″,7″). ^{13}C-NMR: Table S1.

Compound 10：11α-O-2-methylbutyryl-12β-O-tigloyltenacigenin B

Colorless needle crystals (methanol), ^{1}H-NMR($CDCl_3$): δ_H0.80 (3H, t, J=7.2 Hz, 4′-CH_3), 0.98 (3H, d, J=6.6 Hz, 5′-CH_3), 1.07 (3H, s,19-CH_3), 1.09 (3H, s, 18-CH_3), 1.75 (3H, br s, 5″-CH_3), 1.76 (3H, d, J=6.0 Hz, 4″-CH_3), 2.23 (3H, s, 21-CH_3), 2.92 (1H, br d, J=7.2 Hz, H-17β), 3.60 (1H, m, H-3), 5.05 (1H, d, J=10.2 Hz, H-12α), 5.42 (1H, t, J=10.2Hz, H-11β), 6.80 (1H, q, J=5.4 Hz, H-3″). ^{13}C-NMR: Table S2.

Compound 11：Marsdenoside B

Colorless needle crystals (methanol), ^{1}H-NMR ($CDCl_3$): δ_H1.07 (3H, s,19-CH_3), 1.12 (3H, s, 18-CH_3), 1.26 (3H, d, J=6.0 Hz, Allo-6-CH_3), 1.33 (3H, d, J=4.8 Hz,

Ole-6-CH_3), 1.48 (1H, m, Ole-Ha-2), 1.67 (6H, br s, 5′,5″-CH_3), 1.69, 1.71 (each 3H, d, *J*=7.2 Hz, 4′,4″-CH_3), 2.04 (1H, d, *J*=10.2 Hz, H-9), 2.22 (3H, s, 21-CH_3), 2.31 (1H, dd, *J*=12.5, 2.7 Hz, Ole-He-2), 2.93 (1H, br d, *J*=7.2Hz, H-17β), 3.19 (1H, dd, *J*=9.6, 3.0 Hz, Allo-H-4), 3.34 (1H, m, Ole-H-5), 3.38 (3H, s, Ole-3-OCH_3), 3.39 (1H, m, Ole-H-3), 3.49 (1H, dd, *J*=7.8, 2.4 Hz, Allo-H-2), 3.58 (1H, m, Allo-H-5), 3.63 (1H, m, H-3), 3.66 (3H, s, Allo-3-OCH_3), 3.79 (1H, t, *J*=2.7 Hz, Allo-H-3), 4.58 (1H, dd, *J*=9.6, 1.6 Hz, Ole-H-1),4.79 (1H, d, *J*=7.8 Hz, Allo-H-1), 5.05 (1H, d, *J*=10.2 Hz, H-12α), 5.47 (1H, t, *J*=10.2 Hz, H-11β), 6.67, 6.71 (each 1H, qq, *J*=7.2, 1.2 Hz, H-3′,3″). ^{13}C-NMR: Table S2, S3.

Compound 12：11α, 12β-O, O-ditigloyl-17β-tenacigenin B

Colorless needle crystals (methanol), ^{1}H- NMR (MeOD)：δ_{H}1.04 (3H, s, 19-CH_3), 1.09 (3H, s, 18-CH_3), 1.67 (6H, br s, 5′,5″-CH_3), 1.72, 1.73 (each 3H, d, *J*=7.2 Hz, 4′,4″-CH_3), 2.00 (3H, s, 21-CH_3), 5.05(1H, d, *J*=10.2 Hz, 12-H), 5.33 (1H, t, *J*=10.2 Hz, 11-H), 6.70, 6.71 (each 1H, qq, *J*=6.6, 1.2 Hz, H-3′, 3″). ^{13}C-NMR: Table S2.

Compound 13：11-α-O-2-Methy1butyryl-l2β-O-benzoyltenacigenin B

White powder (methanol), ^{1}H-NMR ($CDCl_3$): δ_{H}0.56(3H, t, *J*=7.2 Hz, 4′-CH_3), 0.86(3H, d, *J*=6.6 Hz, 5′-CH_3), 1.10 (3H, s, 19-CH_3), 1.17 (3H, s, 18-CH_3), 2.09 (1H, d, *J*=9.6Hz, H-9), 2.28(3H, s, 21-CH_3), 2.99 (1H, d, *J*=7.8 Hz, H-17β), 3.61 (1H, m, H-3), 5.25 (1H, d, *J*=10.2 Hz, H-12α), 5.55 (1H,t, *J*=10.2 Hz, H-11β),7.43 (2H, t, *J*=7.8 Hz, H-4″,6″), 7.55 (1H, t, *J*=7.2 Hz, H-5″), 7.96 (2H, d, *J*=7.8 Hz, H-3″,7″). ^{13}C-NMR: Table S2.

Compound 14：Cimigenol

White powder (methanol)，^{1}H- NMR (MeOD): δ_{H} 3.23 (1H, dd, *J*=13.2, 4.8 Hz, H-3), 0.40 (1H, d, *J*=5.4 Hz, H-19α), 0.64 (1H, d, *J*=4.8 Hz, H-19β), 4.29 (1H, d, *J*=10.8 Hz, H-23), 3.89 (1H, s, H-15), 0.82 (3H, s, 30-CH_3), 0.89 (3H, d, *J*=7.8 Hz, 21-CH_3), 0.96 (3H, s, 18-CH_3), 1.07 (3H, s, 26-CH_3), 1.12 (6H, br s, 28, 29-CH_3), 1.14 (3H, s, 27-CH_3), 3.45 (1H, s, H-24); ^{13}C-NMR (MeOD): δc33.5 (C-1), 32.5 (C-2), 79.5 (C-3), 41.6 (C-4), 48.5 (C-5), 22.2 (C-6), 27.4 (C-7), 49.9 (C-8), 21.2 (C-9), 27.5 (C-10), 26.1 (C-11), 34.9 (C-12), 42.7 (C-13), 48.0 (C-14), 81.2 (C-15), 112.7 (C-16), 60.6 (C-17), 20.0 (C-18), 31.3 (C-19), 24.9 (C-20), 19.9 (C-21), 39.0 (C-22), 73.0 (C-23),

90.9 (C-24), 72.6 (C-25), 27.9 (C-26), 26.1 (C-27), 11.8 (C-28), 25.4 (C-29), 14.7 (C-30).

Compound 17：12β-O-tigloyltenacigenin A

White powder (methanol), ^{1}H-NMR (MeOD): δ_H 1.06 (3H, S, 19-CH_3), 1.09 (3H, s, 18-CH_3), 1.19 (3H, s, 21-CH_3), 1.81 (3H, d, *J*=6.6 Hz, 4′-CH_3), 1.83 (3H, s, 5′-CH_3), 3.50 (1H, m, H-3), 4.38 (1H, dd, *J*=1.8, 3.6 Hz，H-11β), 5.06 (1H, d, *J*=3.6 Hz，H-12α), 6.98 (1H, dd, *J*=1.2, 7.2 Hz, H-3′). ^{13}C-NMR: Table S2.

Compound 19：Tenacissoside H

White powder (methanol), ^{1}H-NMR ($CDCl_3$): δ_H 0.82 (3H, t, *J*=7.6 Hz, 4′-CH_3), 1.01 (3H, d, *J*=7.2 Hz, 5′-CH_3), 1.12 (3H, s, 19-CH_3), 1.19(3H, s, 18-CH_3), 1.75 (3H, d, *J*=6.6 Hz, Allo-6-CH_3), 1.57 (3H, d, *J*=6.0 Hz, Ole-6-CH_3), 2.07 (3H, s, 2″-CH_3), 2.26 (3H, s, 21-CH_3), 2.91 (1H, br d, *J*=6.6 Hz, H-17β), 3.85 (3H, s, Allo-3-OCH_3), 3.56 (3H, s, Ole-3-OCH_3), 4.82 (1H, dd, *J*=8.4, 1.6 Hz, Ole-H-1), 5.12 (1H, d, *J*=7.8 Hz, Allo-H-1), 5.36 (1H, d, *J*=10.1 Hz, H-12α), 5.60 (1H, t, *J*=10.2 Hz, H-11β). ^{13}C-NMR: Table S2, S3.

Compound 20：11α-O-2-methylbutyryl-12β-O-acetyltenacigenin B

White powder (methanol), ^{1}H-NMR ($CDCl_3$): δ_H 0.82 (3H, t, *J*=7.6 Hz, 4′-CH_3), 1.01 (3H, d, *J*=7.2 Hz, 5′-CH_3), 1.12 (3H, s, 19-CH_3), 1.19(3H, s, 18-CH_3), 2.09 (3H, s, 2″-CH_3), 2.25 (3H, s, 21-CH_3), 2.91 (1H, br d, *J*=6.6 Hz, H-17β), 5.36 (1H, d, *J*=10.2 Hz, H-12α), 5.61 (1H, t, *J*=10.2 Hz, H-11β). ^{13}C-NMR: Table S2.
