# Supplementary material for: A Combined Phytochemistry and Network Pharmacology Approach to Reveal Potential Anti-NSCLC Effective Substances and Mechanisms in Marsdenia tenacissima (Roxb.) Moon (Stem)
Source: Front Pharmacol. 2021 Apr 29;12:518406. doi: 10.3389/fphar.2021.518406 (PMC8117745; doi:10.3389/fphar.2021.518406)
Supplement: Supplementary file 1 [file datasheet1.zip › Data Sheet/Supplementary Material/Table S1.pdf]

Table S1. ^{13}C-NMR data of the aglycone of Compound 1-4 and 7-9(1-4 in MeOD, 7-9 in CDCl$_3$)

C	1	2	3	4	7	8	9
1	38.8	38.7	38.8	38.7	37.8	37.5	37.3
2	30.3	30.3	30.3	30.2	29.0	29.0	31.4
3	70.8	70.1	70.8	70.2	76.2	76.2	70.6
4	38.8	38.7	38.8	38.7	34.5	34.8	38.3
5	45.1	45.1	45.2	45.2	43.9	43.9	44.1
6	27.7	27.7	27.7	27.7	26.6	26.7	26.7
7	32.9	32.9	32.9	32.9	31.8	31.8	31.8
8	68.2	68.2	68.2	68.2	66.8	66.8	66.9
9	52.8	52.6	52.8	52.6	51.1	51.1	51.2
10	40.2	39.1	39.0	39.1	39.1	39.1	38.9
11	70.8	70.2	70.8	70.2	68.5	68.7	68.8
12	76.0	75.7	76.0	75.6	76.1	74.7	75.5
13	47.0	47.2	47.0	47.2	46.1	46.0	46.1
14	73.1	73.0	73.1	73.0	71.4	71.4	71.5
15	27.7	27.8	27.7	27.8	26.8	26.6	26.7
16	26.0	25.9	26.0	25.9	25.0	25.0	25.1
17	60.9	61.1	60.9	61.1	60.1	60.0	59.8
18	16.9	17.0	16.9	17.0	16.8	16.8	16.6
19	13.2	13.3	13.3	13.3	12.7	12.7	12.7
20	213.1	213.1	213.1	213.1	210.9	210.8	211.1
21	30.3	31.9	31.8	31.9	30.0	30.0	30.3
	Bz	Tig	Bz	Tig	Bu	Bu	Tig
1′	167.6	168.7	167.4	168.6	175.7	175.6	167.4
2′	131.3	130.0	131.3	129.1	41.2	41.3	128.4
3′	130.6	139.9	130.5	139.8	25.8	25.9	129.4
4′	129.8	12.0	129.8	12.0	11.4	11.7	11.6

5′	134.7	14.5	134.6	14.5	15.1	15.2	15.2
6′	129.8		129.8				
7′	130.6		130.5				
	Ac	Ac	Ac	Ac	Bz	Tig	Bz
1″	172.2	172.2	172.1	172.2	166.1	167.4	166.1
2″	20.3	20.5	20.3	21.6	129.4	128.0	133.1
3″					129.8	138.6	129.7
4″					128.5	11.9	128.3
5″					133.3	14.5	138.1
6″					128.5		128.3
7″					129.8		129.7
