# Supplementary material for: A Combined Phytochemistry and Network Pharmacology Approach to Reveal Potential Anti-NSCLC Effective Substances and Mechanisms in Marsdenia tenacissima (Roxb.) Moon (Stem)
Source: Front Pharmacol. 2021 Apr 29;12:518406. doi: 10.3389/fphar.2021.518406 (PMC8117745; doi:10.3389/fphar.2021.518406)

Table S2. ^{13}C-NMR data of the aglycone of Compound 10-13, 15, 17, 19 and 20

(12 and 17 in MeOD, others in $CDCl_3$)

C	10	11	12	13	17	19	20
1	37.6	37.5	38.5	37.6	40.2	37.6	37.5
2	31.2	29.8	31.5	31.2	31.3	29.9	31.0
3	70.5	76.4	71.0	70.5	74.5	76.1	70.4
4	38.4	34.9	38.9	38.3	38.0	34.7	38.3
5	44.0	44.1	45.3	44.0	47.6	43.9	44.0
6	26.6	29.4	28.0	26.7	29.0	26.8	26.6
7	31.8	31.9	33.2	31.8	34.8	31.8	31.7
8	66.9	66.8	67.7	66.9	79.7	66.8	66.7
9	51.0	51.2	53.3	51.1	59.4	51.1	51.0
10	38.9	39.0	40.3	39.0	36.5	39.0	38.7
11	68.6	68.9	69.5	68.5	69.8	68.5	68.4
12	74.6	74.7	80.2	75.4	71.8	75.2	75.0
13	46.0	46.0	45.3	46.1	45.6	45.8	45.7
14	71.4	71.5	72.9	71.4	81.3	71.4	71.3
15	26.6	26.7	28.0	26.6	35.4	26.6	26.5
16	24.9	25.0	27.2	25.0	23.8	25.0	24.9
17	60.0	59.8	62.2	60.0	56.7	60.2	60.0
18	16.8	16.6	14.5	16.8	17.4	16.8	16.7
19	12.8	12.6	13.2	12.8	17.2	12.7	12.6
20	210.8	211.1	211.1	210.8	101.2	210.6	210.6
21	30.0	30.3	31.8	30.0	23.6	29.7	29.6
	Bu	Tig	Tig	Bu	Tig	Bu	Bu
1′	175.6	167.3	168.6	175.7	169.6	175.6	175.6
2′	41.3	127.8	129.5	41.2	129.7	41.3	41.3
3′	25.9	137.8	139.6	25.8	140.3	26.0	26.1

4′	11.7	11.8	11.8	11.5	12.2	11.7	11.7
5′	15.2	14.4	14.4	15.1	14.6	15.3	15.3
6′							
7′							
	Tig	Tig	Tig	Bz		Ac	Ac
1″	167.4	167.5	168.8	166.1		170.8	170.8
2″	128.0	128.7	129.6	129.4		20.8	20.7
3″	138.6	138.1	139.9	129.8			
4″	11.9	11.8	11.9	128.5			
5″	14.5	14.4	14.5	133.3			
6″				128.5			
7″				129.8			

Note: Substituent structure

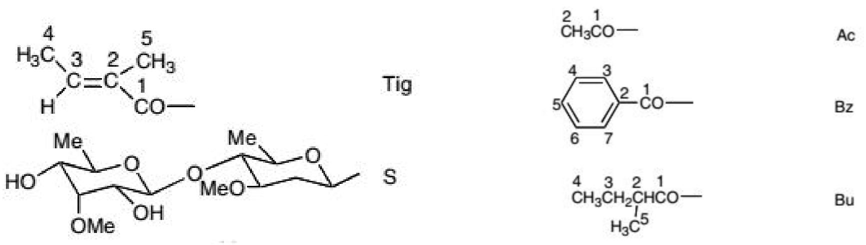

Supplement: Supplementary file 1 [file datasheet1.zip › Data Sheet/Supplementary Material/Table S2.pdf]
