# Supplementary material for: A Combined Phytochemistry and Network Pharmacology Approach to Reveal Potential Anti-NSCLC Effective Substances and Mechanisms in Marsdenia tenacissima (Roxb.) Moon (Stem)
Source: Front Pharmacol. 2021 Apr 29;12:518406. doi: 10.3389/fphar.2021.518406 (PMC8117745; doi:10.3389/fphar.2021.518406)
Supplement: Supplementary file 1 [file datasheet1.zip › Data Sheet/Supplementary Material/Table S3.pdf]

Table S3. ^{13}C-NMR data of the sugar moieties of Compound 1, 2, 7, 8, 11 and 19 (1 and 2 in MeOD, others in $CDCl_3$)

S(Ole)	1	2	7	8	11	19
1	98.6	98.6	96.8	96.8	96.9	96.8
2	37.8	37.9	36.1	36.1	36.1	36.1
3	80.5	80.5	78.8	78.8	78.8	78.8
4	83.9	83.8	79.0	79.0	79.0	79.0
5	72.5	72.5	71.7	71.4	71.4	71.6
6	18.2	18.2	18.6	18.6	18.6	18.6
3-O-Me	57.4	57.4	55.6	55.6	55.6	55.6
S(Allo)						
1	102.2	102.2	99.1	99.1	99.1	99.1
2	71.2	71.2	71.7	71.8	71.7	71.7
3	84.0	83.9	81.0	81.0	81.0	81.0
4	73.6	73.6	72.8	72.8	72.8	72.8
5	70.8	70.1	71.3	71.3	71.2	71.3
6	18.8	18.8	17.9	17.9	17.9	17.9
3-O-Me	62.5	62.5	61.9	62.0	61.9	61.9
