# Supplementary material for: A Combined Phytochemistry and Network Pharmacology Approach to Reveal Potential Anti-NSCLC Effective Substances and Mechanisms in Marsdenia tenacissima (Roxb.) Moon (Stem)
Source: Front Pharmacol. 2021 Apr 29;12:518406. doi: 10.3389/fphar.2021.518406 (PMC8117745; doi:10.3389/fphar.2021.518406)
Supplement: Supplementary file 1 [file datasheet1.zip › Data Sheet/Supplementary Material/Figure S1.doc]

**Figure S1. ^1^H-NMR, ^13^C-NMR, HMBC, HSQC and NOESY Spectrum of Compound 15**

^1^H-NMR Spectrum of Compound 15

^13^C-NMR Spectrum of Compound 15

HMBC Spectrum of Compound 15 HSQC Spectrum of Compound 15

NOESY Spectrum of Compound 15
