# Supplementary material for: A Combined Phytochemistry and Network Pharmacology Approach to Reveal Potential Anti-NSCLC Effective Substances and Mechanisms in Marsdenia tenacissima (Roxb.) Moon (Stem)
Source: Front Pharmacol. 2021 Apr 29;12:518406. doi: 10.3389/fphar.2021.518406 (PMC8117745; doi:10.3389/fphar.2021.518406)
Supplement: Supplementary file 1 [file datasheet1.zip › Data Sheet/Supplementary Material/Figure S2.doc]

**Figure S2. ^1^H-NMR, ^13^C-NMR, 1H-1H COSY ,HMBC, HSQC and NOESY Spectrum of Compound 18**

^1^H-NMR Spectrum of Compound 18

^13^C-NMR Spectrum of Compound 18

^1^H-^1^H COSY Spectrum of Compound 18

HMBC Spectrum of Compound 18

HSQC Spectrum of Compound 18

NOESY Spectrum of Compound 18
