# Supplementary material for: A Combined Phytochemistry and Network Pharmacology Approach to Reveal Potential Anti-NSCLC Effective Substances and Mechanisms in Marsdenia tenacissima (Roxb.) Moon (Stem)
Source: Front Pharmacol. 2021 Apr 29;12:518406. doi: 10.3389/fphar.2021.518406 (PMC8117745; doi:10.3389/fphar.2021.518406)
Supplement: Supplementary file 1 [file datasheet1.zip › Data Sheet/Supplementary Material/Table S1.doc]

**Table S1. ^13^C-NMR data of the aglycone of Compound 1-4 and 7-9(1-4 in MeOD, 7-9 in CDCl_3_)**

| C | 1 | 2 | 3 | 4 | 7 | 8 | 9 |
| --- | --- | --- | --- | --- | --- | --- | --- |
| 1 | 38.8 | 38.7 | 38.8 | 38.7 | 37.8 | 37.5 | 37.3 |
| 2 | 30.3 | 30.3 | 30.3 | 30.2 | 29.0 | 29.0 | 31.4 |
| 3 | 70.8 | 70.1 | 70.8 | 70.2 | 76.2 | 76.2 | 70.6 |
| 4 | 38.8 | 38.7 | 38.8 | 38.7 | 34.5 | 34.8 | 38.3 |
| 5 | 45.1 | 45.1 | 45.2 | 45.2 | 43.9 | 43.9 | 44.1 |
| 6 | 27.7 | 27.7 | 27.7 | 27.7 | 26.6 | 26.7 | 26.7 |
| 7 | 32.9 | 32.9 | 32.9 | 32.9 | 31.8 | 31.8 | 31.8 |
| 8 | 68.2 | 68.2 | 68.2 | 68.2 | 66.8 | 66.8 | 66.9 |
| 9 | 52.8 | 52.6 | 52.8 | 52.6 | 51.1 | 51.1 | 51.2 |
| 10 | 40.2 | 39.1 | 39.0 | 39.1 | 39.1 | 39.1 | 38.9 |
| 11 | 70.8 | 70.2 | 70.8 | 70.2 | 68.5 | 68.7 | 68.8 |
| 12 | 76.0 | 75.7 | 76.0 | 75.6 | 76.1 | 74.7 | 75.5 |
| 13 | 47.0 | 47.2 | 47.0 | 47.2 | 46.1 | 46.0 | 46.1 |
| 14 | 73.1 | 73.0 | 73.1 | 73.0 | 71.4 | 71.4 | 71.5 |
| 15 | 27.7 | 27.8 | 27.7 | 27.8 | 26.8 | 26.6 | 26.7 |
| 16 | 26.0 | 25.9 | 26.0 | 25.9 | 25.0 | 25.0 | 25.1 |
| 17 | 60.9 | 61.1 | 60.9 | 61.1 | 60.1 | 60.0 | 59.8 |
| 18 | 16.9 | 17.0 | 16.9 | 17.0 | 16.8 | 16.8 | 16.6 |
| 19 | 13.2 | 13.3 | 13.3 | 13.3 | 12.7 | 12.7 | 12.7 |
| 20 | 213.1 | 213.1 | 213.1 | 213.1 | 210.9 | 210.8 | 211.1 |
| 21 | 30.3 | 31.9 | 31.8 | 31.9 | 30.0 | 30.0 | 30.3 |
|  | Bz | Tig | Bz | Tig | Bu | Bu | Tig |
| 1′ | 167.6 | 168.7 | 167.4 | 168.6 | 175.7 | 175.6 | 167.4 |
| 2′ | 131.3 | 130.0 | 131.3 | 129.1 | 41.2 | 41.3 | 128.4 |
| 3′ | 130.6 | 139.9 | 130.5 | 139.8 | 25.8 | 25.9 | 129.4 |
| 4′ | 129.8 | 12.0 | 129.8 | 12.0 | 11.4 | 11.7 | 11.6 |
| 5′ | 134.7 | 14.5 | 134.6 | 14.5 | 15.1 | 15.2 | 15.2 |
| 6′ | 129.8 |  | 129.8 |  |  |  |  |
| 7′ | 130.6 |  | 130.5 |  |  |  |  |
|  | Ac | Ac | Ac | Ac | Bz | Tig | Bz |
| 1′′ | 172.2 | 172.2 | 172.1 | 172.2 | 166.1 | 167.4 | 166.1 |
| 2′′ | 20.3 | 20.5 | 20.3 | 21.6 | 129.4 | 128.0 | 133.1 |
| 3′′ |  |  |  |  | 129.8 | 138.6 | 129.7 |
| 4′′ |  |  |  |  | 128.5 | 11.9 | 128.3 |
| 5′′ |  |  |  |  | 133.3 | 14.5 | 138.1 |
| 6′′ |  |  |  |  | 128.5 |  | 128.3 |
| 7′′ |  |  |  |  | 129.8 |  | 129.7 |
