# Supplementary material for: A Combined Phytochemistry and Network Pharmacology Approach to Reveal Potential Anti-NSCLC Effective Substances and Mechanisms in Marsdenia tenacissima (Roxb.) Moon (Stem)
Source: Front Pharmacol. 2021 Apr 29;12:518406. doi: 10.3389/fphar.2021.518406 (PMC8117745; doi:10.3389/fphar.2021.518406)
Supplement: Supplementary file 1 [file datasheet1.zip › Data Sheet/Supplementary Material/Table S2.doc]

**Table S2. ^13^C-NMR data of the aglycone of Compound 10-13, 15, 17, 19 and 20**

**(12 and 17 in MeOD, others in CDCl_3_)**

| C | 10 | 11 | 12 | 13 | 17 | 19 | 20 |
| --- | --- | --- | --- | --- | --- | --- | --- |
| 1 | 37.6 | 37.5 | 38.5 | 37.6 | 40.2 | 37.6 | 37.5 |
| 2 | 31.2 | 29.8 | 31.5 | 31.2 | 31.3 | 29.9 | 31.0 |
| 3 | 70.5 | 76.4 | 71.0 | 70.5 | 74.5 | 76.1 | 70.4 |
| 4 | 38.4 | 34.9 | 38.9 | 38.3 | 38.0 | 34.7 | 38.3 |
| 5 | 44.0 | 44.1 | 45.3 | 44.0 | 47.6 | 43.9 | 44.0 |
| 6 | 26.6 | 29.4 | 28.0 | 26.7 | 29.0 | 26.8 | 26.6 |
| 7 | 31.8 | 31.9 | 33.2 | 31.8 | 34.8 | 31.8 | 31.7 |
| 8 | 66.9 | 66.8 | 67.7 | 66.9 | 79.7 | 66.8 | 66.7 |
| 9 | 51.0 | 51.2 | 53.3 | 51.1 | 59.4 | 51.1 | 51.0 |
| 10 | 38.9 | 39.0 | 40.3 | 39.0 | 36.5 | 39.0 | 38.7 |
| 11 | 68.6 | 68.9 | 69.5 | 68.5 | 69.8 | 68.5 | 68.4 |
| 12 | 74.6 | 74.7 | 80.2 | 75.4 | 71.8 | 75.2 | 75.0 |
| 13 | 46.0 | 46.0 | 45.3 | 46.1 | 45.6 | 45.8 | 45.7 |
| 14 | 71.4 | 71.5 | 72.9 | 71.4 | 81.3 | 71.4 | 71.3 |
| 15 | 26.6 | 26.7 | 28.0 | 26.6 | 35.4 | 26.6 | 26.5 |
| 16 | 24.9 | 25.0 | 27.2 | 25.0 | 23.8 | 25.0 | 24.9 |
| 17 | 60.0 | 59.8 | 62.2 | 60.0 | 56.7 | 60.2 | 60.0 |
| 18 | 16.8 | 16.6 | 14.5 | 16.8 | 17.4 | 16.8 | 16.7 |
| 19 | 12.8 | 12.6 | 13.2 | 12.8 | 17.2 | 12.7 | 12.6 |
| 20 | 210.8 | 211.1 | 211.1 | 210.8 | 101.2 | 210.6 | 210.6 |
| 21 | 30.0 | 30.3 | 31.8 | 30.0 | 23.6 | 29.7 | 29.6 |
|  | Bu | Tig | Tig | Bu | Tig | Bu | Bu |
| 1′ | 175.6 | 167.3 | 168.6 | 175.7 | 169.6 | 175.6 | 175.6 |
| 2′ | 41.3 | 127.8 | 129.5 | 41.2 | 129.7 | 41.3 | 41.3 |
| 3′ | 25.9 | 137.8 | 139.6 | 25.8 | 140.3 | 26.0 | 26.1 |
| 4′ | 11.7 | 11.8 | 11.8 | 11.5 | 12.2 | 11.7 | 11.7 |
| 5′ | 15.2 | 14.4 | 14.4 | 15.1 | 14.6 | 15.3 | 15.3 |
| 6′ |  |  |  |  |  |  |  |
| 7′ |  |  |  |  |  |  |  |
|  | Tig | Tig | Tig | Bz |  | Ac | Ac |
| 1′′ | 167.4 | 167.5 | 168.8 | 166.1 |  | 170.8 | 170.8 |
| 2′′ | 128.0 | 128.7 | 129.6 | 129.4 |  | 20.8 | 20.7 |
| 3′′ | 138.6 | 138.1 | 139.9 | 129.8 |  |  |  |
| 4′′ | 11.9 | 11.8 | 11.9 | 128.5 |  |  |  |
| 5′′ | 14.5 | 14.4 | 14.5 | 133.3 |  |  |  |
| 6′′ |  |  |  | 128.5 |  |  |  |
| 7′′ |  |  |  | 129.8 |  |  |  |

Note: Substituent structure
