# Supplementary material for: A Combined Phytochemistry and Network Pharmacology Approach to Reveal Potential Anti-NSCLC Effective Substances and Mechanisms in Marsdenia tenacissima (Roxb.) Moon (Stem)
Source: Front Pharmacol. 2021 Apr 29;12:518406. doi: 10.3389/fphar.2021.518406 (PMC8117745; doi:10.3389/fphar.2021.518406)
Supplement: Supplementary file 1 [file datasheet1.zip › Data Sheet/Supplementary Material/Table S3.doc]

**Table S3. ^13^C-NMR data of the sugar moieties of Compound 1, 2, 7, 8, 11 and 19**

**(1 and 2 in MeOD, others in CDCl_3_)**

| S(Ole) | 1 | 2 | 7 | 8 | 11 | 19 |
| --- | --- | --- | --- | --- | --- | --- |
| 1 | 98.6 | 98.6 | 96.8 | 96.8 | 96.9 | 96.8 |
| 2 | 37.8 | 37.9 | 36.1 | 36.1 | 36.1 | 36.1 |
| 3 | 80.5 | 80.5 | 78.8 | 78.8 | 78.8 | 78.8 |
| 4 | 83.9 | 83.8 | 79.0 | 79.0 | 79.0 | 79.0 |
| 5 | 72.5 | 72.5 | 71.7 | 71.4 | 71.4 | 71.6 |
| 6 | 18.2 | 18.2 | 18.6 | 18.6 | 18.6 | 18.6 |
| 3-O-Me | 57.4 | 57.4 | 55.6 | 55.6 | 55.6 | 55.6 |
| S(Allo) |  |  |  |  |  |  |
| 1 | 102.2 | 102.2 | 99.1 | 99.1 | 99.1 | 99.1 |
| 2 | 71.2 | 71.2 | 71.7 | 71.8 | 71.7 | 71.7 |
| 3 | 84.0 | 83.9 | 81.0 | 81.0 | 81.0 | 81.0 |
| 4 | 73.6 | 73.6 | 72.8 | 72.8 | 72.8 | 72.8 |
| 5 | 70.8 | 70.1 | 71.3 | 71.3 | 71.2 | 71.3 |
| 6 | 18.8 | 18.8 | 17.9 | 17.9 | 17.9 | 17.9 |
| 3-O-Me | 62.5 | 62.5 | 61.9 | 62.0 | 61.9 | 61.9 |
